# Why cost reduction alone cannot deliver bankable carbon dioxide removal

**DOI:** 10.1016/j.isci.2026.117702

**Published:** 2026-09-30

**Authors:** Julian Henry Smart, Niall Mac Dowell

**Affiliations:** 1 Centre for Environmental Policy, Imperial College London, 16-18 Prince’s Gardens, South Kensington, London SW7 1NE, UK; 2 The Sargent Centre for Process Systems Engineering, Imperial College London, South Kensington, London SW7 2AZ, UK

**Keywords:** Energy engineering, Energy sustainability, Energy systems, Economics

## Abstract

Carbon dioxide removal (CDR) is indispensable for net-zero, yet CDR projects remain unbankable for institutional lenders. Using a stochastic project-finance model in which financing terms follow from simulated cash flows rather than being assumed, we examine when BECCS and DAC become bankable. Under unhedged conditions bankability is effectively zero, with lender coverage failing across most of the financing space and equity hurdles met in less than 0.1%. Contrary to the emphasis on cost reduction, credit-worthy offtake is the primary determinant. Learning alone lowers the levelized cost of removal by up to 38%, yet leaves projects unbankable, whereas offtake raises BECCS bankability to 43%, or 57% with learning. DAC requires offtake above US$2,500 per tCO_2_ and remains marginally bankable, reflecting sole dependence on carbon revenues. An electricity-anchored BECCS pathway can reach investment grade without fully contracted carbon revenues. These results show where revenue support must be targeted to mobilize institutional capital.

## Highlights

• Merchant BECCS and DAC are unbankable; equity hurdles met in <0.1% of simulations
• Capital cost cuts of 40% lower removal cost by 38% yet leave bankability near zero
• Creditworthy offtake at US$350–400 per tCO_2_ lifts BECCS to investment grade
• Learning plus offtake cuts BECCS removal cost to US$161/tCO_2_ at 57% bankability

## Graphical abstract

**Figure:**
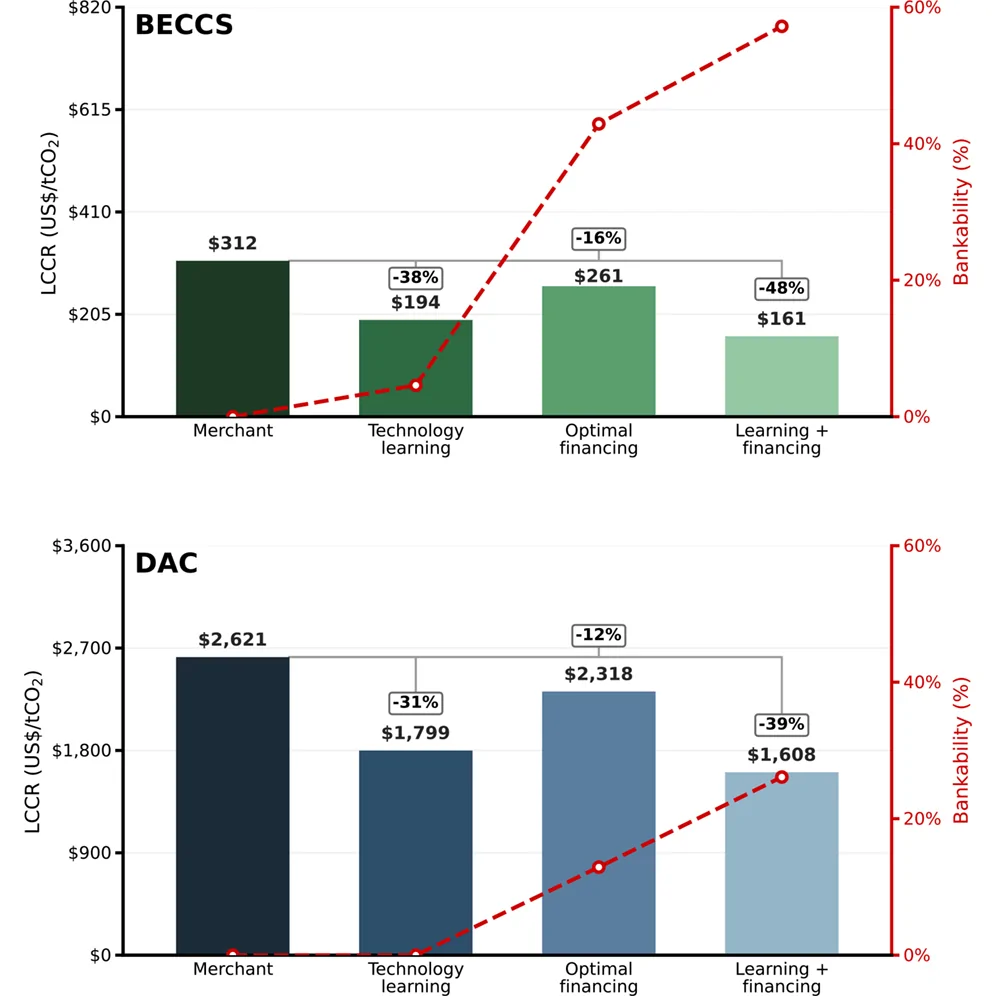

## Introduction

Carbon dioxide removal (CDR) is increasingly recognized as indispensable for achieving mid-century climate targets. Integrated assessment models indicate a need for 7 to 9 Gt CO_2_ per year of removals by 2050.^1^ National strategies and dedicated removal budgets,^2^ such as the United Kingdom’s Greenhouse Gas Removal (GGR) program, are now being developed and target tens of megatons removal capacity per year.^3^ However, despite this increasing confidence in CDR, its commercial deployment remains negligible.^4^ Early projects have relied predominantly on grant funding, philanthropic funding, buyers’ clubs, or bespoke offtake arrangements rather than project finance.^5^ Consistent with this, only a small number of debt-financed CDR transactions have been publicly disclosed, including recent first-of-a-kind (FOAK) structures by UNDO^6^ for enhanced rock weathering (ERW) and a carbon capture and storage (CCS) dedicated bond issuance linked to CO_2_ transport and storage (T&S) infrastructure in the Port of Rotterdam.^7^ Together, this suggests that, alongside technological uncertainty and cost challenges, financing conditions are not yet compatible with mobilizing private capital at scale. More specifically, few projects can be structured to satisfy lender coverage requirements under downside uncertainty implied by observed market volatility, leaving access to debt capital markets limited.

Considerable work has been conducted using technoeconomic assessment (TEA) and life cycle assessment (LCA) methodologies to estimate the prospective technology costs and system needs for CDR pathways, including BECCS, DAC, biochar, and ERW. This work has been essential in establishing the main drivers of cost and developing a first-order estimate of levelized costs, which can vary by an order of magnitude depending on assumptions and scale. The range of published estimates generally falls within $100–3,000/tCO_2_ depending on pathways and system boundaries.^8^^,^^9^^,^^10^ In addition, learning curve and other cost reduction assumptions are also frequently included in TEA studies, suggesting that costs could be significantly lower in the future, for example, $100–300/tCO_2_ in some mid-century scenarios^3^^,^^11^; however, these projections remain highly uncertain and sensitive to assumptions about scale, supply chains, and operating performance. Other studies have addressed the physical potential of CDR technologies, providing a crucial upper bound on what might be physically delivered absent other frictions.^12^^,^^13^^,^^14^

However, the same literature often treats financing implicitly, typically by using an assumed discount rate or fixed weighted average cost of capital (WACC) with the result that the pathway appears competitive in expectation without consideration of realistic financing constraints. This is useful for direct technology comparison, but it does not provide a basis for determining the ability to finance on repeatable terms under conditions of uncertainty. A single discount rate is not representative of project finance which is underwritten on covenant compliance under downside stress. Large-scale deployment requires capital structures that can withstand volatility and downside risks, because the ability to raise finance is predicated on how these risks are perceived and priced.^15^ Therefore, a technology may be found to be competitive based on levelized costs but still may not attract private capital based on revenues that are short in duration and less contractable.^16^ As such, there is a distinction between low expected CDR costs and the capacity to deploy CDR at scale on bankable terms, and the literature has tended to address the former.

This distinction extends to the discount rate itself. The societal case for large-scale CDR rests on social cost-benefit analysis, where a long-standing debate over the appropriate social discount rate^17^^,^^18^ has converged toward values of around 2%–3%, with declining rates advocated for long horizons.^19^^,^^20^ The choice is consequential for removals, as higher discount rates shift the mitigation burden toward the future and increase the reliance on negative emissions in cost-optimal pathways.^21^ Private capital, however, does not lend at the social discount rate. It prices project-specific risk, and for unproven, merchant-exposed assets the resulting cost of capital is several times the social rate, reaching rates many multiples larger. CDR can therefore pass a social cost-benefit test at the rates society applies yet remain undeliverable at the rates private capital requires.

A small growing set of studies and commentaries has begun to engage directly with the implications of financing beyond the implicit assumption of unlimited access to capital markets. Some academic and gray literature highlights that financing assumptions can materially alter levelized costs. For example, a recent analysis shows that varying the cost of capital between 2.5% and 15%, representative of the US treasury-bill rate and FOAK costs of capital, respectively, can increase the cost of removal by almost 90%.^22^ Some project-level studies have incorporated discounted cash-flow modeling with CDR.^23^^,^^24^ Generally, these studies have relied on deterministic sensitivity analysis or exogenous financing terms rather than modeling the downstream impact of risk and uncertainty on the resulting cost of capital and cost of removal.

A separate strand of risk-aware appraisal applies quantile-based measures such as value-at-risk (VaR) and conditional value-at-risk (CVaR)^25^^,^^26^ to energy investment and planning.^27^ This has recently reached carbon removal through the carbon-at-risk (CaR) measure, a VaR-inspired estimate of the shortfall between contracted removals and realized storage, calibrated to forest carbon and DACCS,^28^ while real options analysis has examined CCS investment under climate policy uncertainty,^29^ robust decision and deep-uncertainty methods have been applied to long-term climate policy and BECCS investment,^30^^,^^31^ and portfolio methods to CDR technology selection.^10^^,^^32^ These approaches characterize downside outcomes rigorously, but address delivered volumes, investment timing, or technology choice rather than financing. Quantile measures have also been embedded in energy system optimization. Three studies using the BENOPTex energy system model to Germany. Two place VaR and CVaR in the objective function, one comparing BECCS and DACCS deployment,^33^ and the other applying both measures to the cost of covering electricity shortfalls, finding that stronger risk aversion increases reliance on dispatchable generation, including fossil plant, and makes BECCS more attractive as a source of dispatchable output that also removes emissions.^34^ The third assesses the combined deployment of technical and natural sinks, with uncertainty addressed through parameter sensitivity.^35^ In a land use application, CVaR has been applied to the net present value of tree planting in the United Kingdom, with the measure taken over the worst tenth of outcomes and alternative removal entered as a hypothetical risk free cost per tonne.^36^ These applications determine which pathways enter a cost optimal system solution, or which planting strategy is preferred on social net present value, neither of which establishes whether an individual project can raise and service debt on the terms available to it (see STAR Methods: relation to VaR and CVaR approaches).

Private-sector rating platforms such as Sylvera and BeZero Carbon have begun developing methodologies to score the integrity and delivery of carbon credits. Their ratings are increasingly used by buyers as signals of credit quality and price risk, with higher ratings linked to lower perceived price volatility.^37^^,^^38^ These ratings speak to the credit quality but do not address the financial bankability of the underlying removal projects. In parallel, policy taxonomies and architectures are emerging—including the UK Greenhouse Gas Removal Business Model (GGRBM),^39^ 45Q under the Inflation Reduction Act (IRA),^40^ and the EU Carbon Removal and Carbon Farming (EU CRCF) framework^41^, that aim to develop CDR integrity and certification, and in the case of the GGRBM, provide contract-based revenue support through carbon contracts for difference (CCfD) with HM Government as an implicit insurer of last resort.^42^^,^^43^^,^^44^ Two parallel streams therefore exist, one quantifying cost reduction pathways for BECCS and DAC, another developing revenue stability frameworks, yet neither has modeled how these channels propagate through project-level financing under uncertainty. While the governance dimension of this financing, including the design of durable offtake frameworks and the political economy of long-term revenue commitments, are being addressed by emerging CDR policy literature, the mechanism linking market design to bankability under uncertainty has not been directly examined through a capital market perspective.

Our work addresses this gap by combining techno-economic modeling with a stochastic project-finance risk-allocation and credit-rating framework. We treat financing endogenously by mapping stochastic cash flows into perceived credit risk and, in turn, the availability and pricing of debt, propagating these conditions into the effective cost of carbon removal and a practical measure of project bankability. We use BECCS and DAC as case studies, selected for their prominence in national plans and their different commercial profiles. By testing alternative market structures and de-risking mechanisms, we examine how financing barriers can be alleviated and under what conditions projects approach investment-grade financing profiles. We find that revenue contractability, not cost reduction, is the primary determinant of bankability because lenders assess credit risk on the downside cash flow distribution rather than expected costs. Capital reductions lower removal costs without improving credit quality, whereas long-term revenues stabilize the downside and unlock leverage. To our knowledge, this is the first study to model CDR bankability through endogenous credit rating under stochastic uncertainty, establishing that revenue de-risking, within observed transaction ranges, is a precondition for cost reduction to be effective at scale. We also show that BECCS can move into an investment-grade envelope when supported by a premium for low-carbon electricity that is comparable to early low-carbon power contracting arrangements.

## Results

We focus the analysis by applying the framework to BECCS and direct air capture (DAC) to quantify how market design and business support translate into perceived credit risk, financing and the effective cost of removal. BECCS and DAC are the focus due to their prominence in national strategies and proposals to integrate removals into emissions trading schemes and related business models, as well as their contrasting commercial profiles. In comparison, pathways such as biochar and ERW currently operate through different market architectures and contracting conventions and are less directly aligned with near-term emissions trading scheme integration and contemporary certification methodologies. While the framework itself is portable to these pathways, applying it requires re-parameterization, and the quantitative results reported here are specific to BECCS and DAC.

All cost, performance and financing inputs follow the calibration described in “STAR Methods: Carbon Dioxide Removal Parameters & Quantification and Statistical Analysis,” using market data where available and analogs from adjacent project-finance sectors where gaps remain. We evaluate three families of market conditions. In the merchant (uncontracted) case, all revenues (the carbon price and electricity for BECCS) and key input costs are fully exposed to market volatility, modeled as stochastic and unhedged over the project’s life with no risk transfer. Risk-transfer (hedged) scenarios stabilize one or more of these variables through a contract, such as a fixed carbon price via a CCfD, a fixed power price via a PPA, or a fixed feedstock price via a procurement contract. Each is parameterized by a coverage, the fraction of exposure hedged (25%–100%), and a strike, the fixed price at which it is contracted; in the model these enter as a reduction in the variance and downside of the corresponding cash-flow component. The fully contracted case is the limiting scenario in which revenue is stabilized at 100% coverage through policy- or contract-backed offtake.

A project is bankable when it can raise finance on acceptable terms; because lenders underwrite on downside rather than expected cash flows, a low-cost technology can remain unbankable if its revenues are too volatile or non-existent. Within each family, policy and contract interventions are represented through their financial effects rather than their legal form. We translate the resulting stochastic cash flows into financing conditions by mapping simulated cash flows into stand-alone credit profiles (SACPs), the project’s implied rating, using S&P’s project rating framework, which we adapt to CDR assets (see STAR Methods). The SACP implies feasible financing terms under each market scenario, which in turn determine the project’s cost of capital and a credit-adjusted levelized cost of carbon removal (LCCR), the net per tonne cost of removal inclusive of financing. Figure 1 summarizes the workflow of the framework.

**Figure 1:**
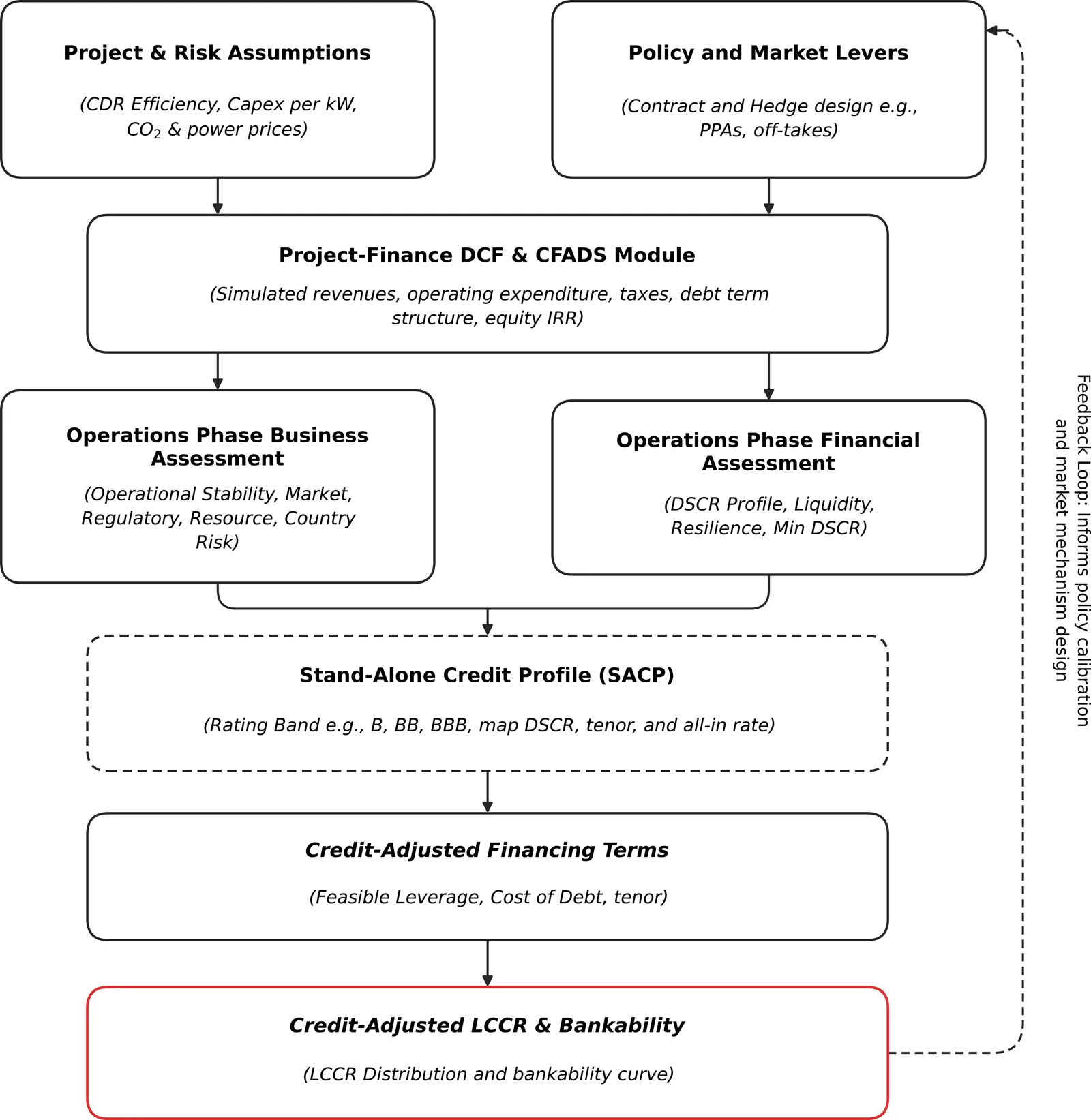
Framework linking stochastic project cash flows to credit rating and financing terms The framework integrates project-finance simulation under uncertainty with credit-based assessment. The framework samples uncertainty in technology performance, costs, and carbon/power prices and feeds a project-finance discounted cash flow (DCF) model that produces cash-flow available for debt servicing (CFADS) i.e., the cash remaining to pay lenders, debt service coverage ratios (DSCRs) i.e., the number of times CFADS covers debt service, and equity internal rate of return (IRR) distributions. These are mapped, via an operations-phase business and financial assessment into a stand-alone credit profile (SACP), which is a reference for the credit risk of the underlying project. These determine feasible leverage, cost of debt and LCCR. Policy and market levers (CfDs, PPAs, long-term offtake) act by reshaping cash-flow risk, improving the SACP, relaxing rating-aligned constraints on DSCR, tenor, and spreads. The resulting credit-adjusted financing terms yield credit-adjusted LCCR and simulated probabilities of bankability. The feedback loop illustrates how policy calibration can target desired expected credit quality (SACP) or cost outcomes (LCCR). See also Tables S6–S11.

Overall, the results for both technologies point to bankability as a prominent constraint on CDR deployment, particularly relative to prevailing carbon pricing environments. Measures that stabilize revenues improve credit quality and expand financing capacity, whereas cost compression primarily reduces LCCR with comparatively limited impact on credit risk. Achieving investment-grade profiles does not require full price coverage in all cases, but it does require sufficiently durable revenue support at strike prices that maintain adequate downside protection. Electricity-anchored business models can also support investment-grade credit risk when buyers are willing to pay a premium for low-carbon electricity, providing an additional contracted revenue base. By contrast, technological learning alone does not render merchant BECCS or DAC financeable unless revenue and key costs are at least partially stabilized.

### Uncontracted CDR: Bankability under unhedged revenues and costs

In the merchant case, both revenues and key input costs are modeled as stochastic and unhedged over the project life, capturing volatility and downside risk across Monte Carlo simulations. Under this uncontracted risk allocation, both BECCS and DAC exhibit sub-investment-grade characteristics, with an implied stand-alone credit profile of B-, which on the standard S&P rating scale (running from CCC up to AAA) sits well below the BBB- threshold that defines investment grade. For each pathway and simulation, we compute the annual cash flow available for debt service (CFADS), which is the cash available to repay lenders, and the debt service coverage ratio (DSCR), which is the number of times that cash covers scheduled debt service, where a value below one implies a shortfall. Using the minimum DSCR over the debt schedule, we classify a configuration as lender feasible if the minimum DSCR exceeds 1.20x, a threshold that is conservative by project financing and energy infrastructure standards but serves to emphasize the findings related to the early commercial state of CDR. Under contracted conditions, the lender hurdle is rating dependent as per Table S1. Equity feasibility is assessed on the same simulation set by computing the levered equity internal rate of return from equity cash flows and applying a 12% hurdle rate as an approximation for the equity risk premium demanded by equity investors in an unproven, policy-dependant asset class without an established performance track record. This is deliberately conservative, given the nascency of CDR. We then conducted a parameter sweep across leverage and tenor combinations and applied a ±500 basis point shift around the endogenously determined base interest rate implied by the merchant credit profile.

Under merchant exposure, lender feasibility is confined to a narrow section of the financing space (Figure 2). For BECCS, DSCR compliance peaks at 95% of simulations at 5% leverage under the most favorable assumptions but declines rapidly as leverage increases and collapses beyond 15% even when tenors are extended. DAC performs worse, with a minimum DSCR pass rate of only 29.4% across the parameter space, reflecting its single revenue stream. In the scenario aligned with B- financing, the endogenously implied merchant debt package of approximately 5-year tenor, low leverage, and 10% interest rates, the DSCR threshold is met in between 46% and 92% of simulations for BECCS and 1.2%–24.2% for DAC. Outside this region, lender feasibility collapses. At 50% debt, DSCR compliance falls to below 6.7% for BECCS and zero for DAC, and at 75% leverage is almost never achieved regardless of technology.

**Figure 2:**
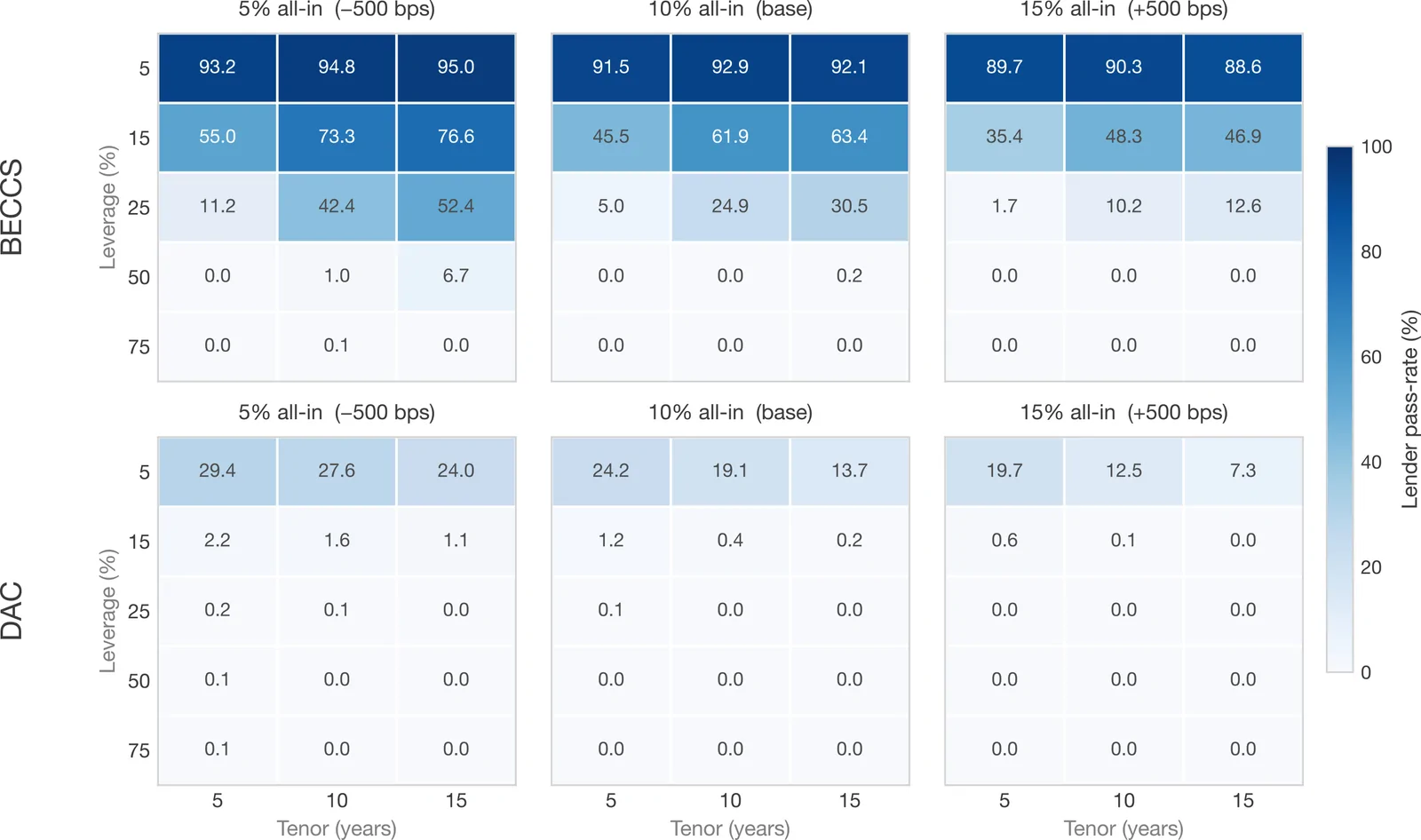
Merchant lender feasibility for BECCS (top) and DAC (bottom) Each cell reports the share of Monte Carlo simulations in which the minimum debt service coverage ratio (DSCR) over the debt schedule meets or exceeds 1.20 (the lender coverage test; that is lenient by project-finance standards, so the pass-rates represent a favorable case). Rows vary by leverage (5%, 15%, 25%, 50%, 75%) and columns vary debt tenor (5, 10, 15 years). Panels correspond to all-in debt rates of 5% (−500 bps), 10% (base), and 15% (+500 bps). Cash flows reflect observable uncertainty with no risk transfer (merchant exposure). Shading encodes the pass-rate on a common 0%–100% scale across both pathways, so DAC’s narrower feasible region is directly comparable. Equity feasibility (levered equity IRR ≥12%) is met in less than 0.1% of simulations in every cell and is therefore omitted. See also Table S1.

Equity feasibility is even more restrictive. With a 12% hurdle rate, equity IRR clears approximately 0% of the time across all leverage-tenor combinations. Even in the most favorable configuration, equity feasibility remains negligible, with the hurdle clearing in only 0.1% of simulations, occurring at the longest tenor and highest leverage tested with the lowest interest rate, and essentially never at other leverage levels. A lower hurdle rate would seldom impact the equity feasibility under merchant conditions due to insufficient equity returns.

Taken together, these results indicate that within this fully unhedged merchant structure, no tested combination of leverage, tenor, and all-in debt rate produce simultaneous lender and equity feasibility with non-trivial probability; interest-rate shifts move debt coverage modestly without changing the overall bankability. Merchant volatility therefore renders unsupported BECCS and DAC unbankable.

### How stabilization mechanisms shift bankability

Given that BECCS and DAC are unbankable under merchant conditions, we examine how stabilizing individual risk drivers shifts project bankability. Figure 3 summarizes how stabilization of each risk driver shifts maximum leverage, debt feasibility, equity feasibility, and ultimately bankability.

**Figure 3:**
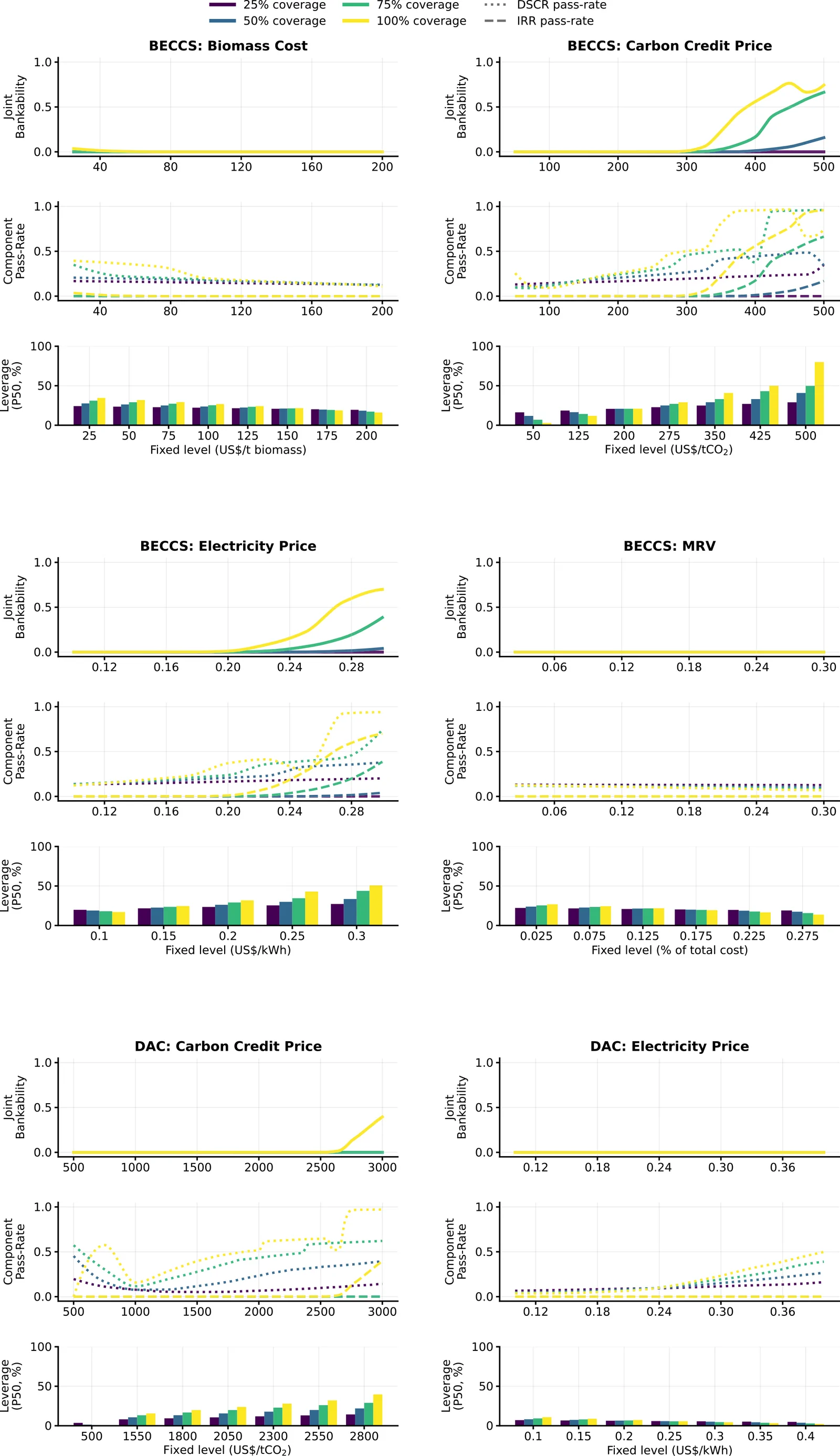
Bankability decomposition under partial and full hedging Coverage levels correspond to 25%–100% stabilization of each respective risk i.e., long-term contracts that secure specific costs or revenues e.g., long-term procurement contracts or offtake agreements. Top panels show joint bankability, the fraction of simulations meeting both the DSCR and equity IRR tests; middle panels show the DSCR (dotted) and IRR (dashed) component pass-rates separately; bottom panels show maximum feasible leverage at the median (P50) across simulations. See also Figures S4, S6, S8, and S9.

Introducing fixed CDR price support restructures cash flow volatility and bankability. For BECCS, bankability remains effectively zero below approximately US$300 per tCO_2_, as lender coverage improves but equity feasibility remains negligible. Above this threshold the response is non-linear. At US$400 per tCO_2_, lender and equity feasibility reach 96% and 56%respectively, with combined bankability of 56%. DAC requires substantially higher prices, reflecting its single revenue stream and capital intensity. Below US$1,000 per tCO_2_ equity feasibility remains zero despite improving lender coverage; only around US$2,500 per tCO_2_ do both lender and equity conditions materially improve. While expensive, transactions in the voluntary carbon market (VCM) have occurred at similar strike prices; in particular the bilateral agreement between Stripe and Heirloom.^45^

Partial revenue support yields limited improvement, but above approximately US$350 per tCO_2_, BECCS bankability increases sharply as downside tails are removed and DSCR compliance becomes robust. While this is likely to be the case for partial coverage with DAC, it is not apparent within our tested offtake price range. A similar pattern is observed for electricity stabilization for BECCS, with bankability increasing significantly only once fixed prices exceed approximately US$250 to US$275 per MWh.

By contrast, input cost stabilization exhibits weaker effects. A long-term biomass procurement contract at US$200 per tonne of biomass increases DSCR stability but achieves lender feasibility in less than 11% of simulations. Even at US$25 per tonne of biomass, with the unit cost below the observed average of US$158 per tonne prior to the 2022 biomass price increase, the probability rises to only 39%, with overall bankability at 3.5%. Long term electricity contracts with DAC shows a similar result. Across all scenarios, the best observed DSCR compliance rate is 50%, with no simulations satisfying the bankability condition. This indicates that input-price stabilization reduces operating costs without suppressing the main driver of project uncertainty, revenue volatility, which imparts a material contribution toward a project’s bankability. T&S and MRV cost stabilization show negligible effects across all cost and coverage levels.

### Investment-grade tipping points for CDR

Institutional debt providers rely on credit ratings to determine feasible leverage, tenor, and spreads. Using project-rating methodologies adapted from S&P, we map simulated cash flows into SACPs, as detailed in Methods S1. In the preceding merchant results, uncontracted exposure maps to sub-investment-grade credit quality, which we summarize using an implied stand-alone credit profile of B-. Here we quantify how movements in this credit profile translate into financing terms and therefore the LCCR.^22^

To quantify the link between credit conditions and removal cost, we compute the project WACC ranges implied by each SACP rating. This leads to WACC values of 10%–12%for B profiles and weaker, far above typical infrastructure WACC of 5%–7%.^46^^,^^47^ By contrast, investment-grade conditions, defined as BBB- and above, provide lower spreads and longer tenors, enabling leverage of 70%–80% and reducing WACC to 6%–8%. Figure 4A shows the relationship between credit rating, leverage, and the cost of capital. Across financing configurations, the relationship between WACC and credit quality is non-linear, reflecting the discontinuous shifts in feasible financing terms across rating thresholds.

**Figure 4:**
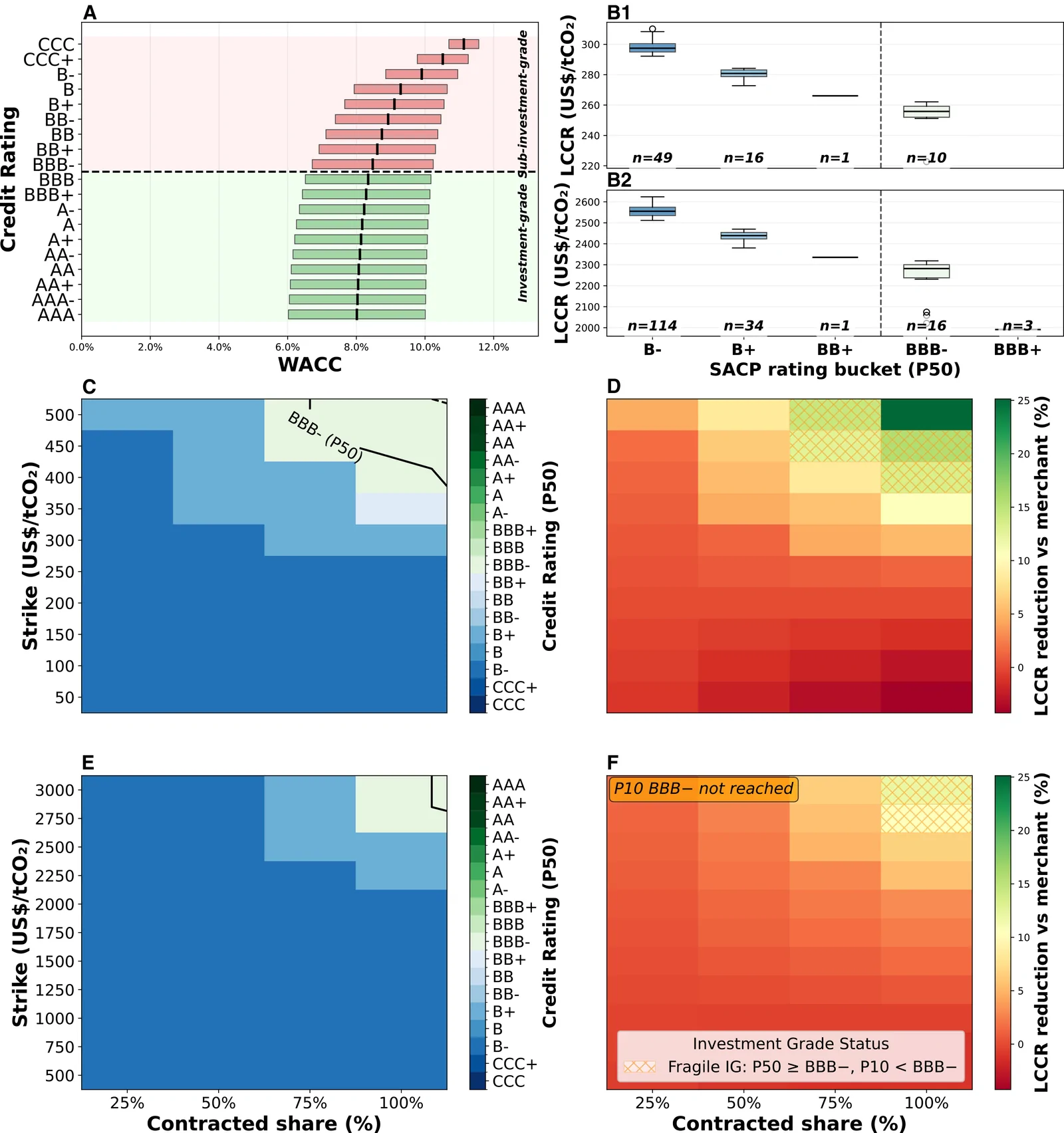
Credit rating and levelized cost response to offtake prices and coverage LCCR heatmaps at base capital cost for CDR offtake and coverage levels (A) cost of capital by credit rating (B) LCCR distributions by credit rating, shown for BECCS (B1) and DAC (B2); boxes span the interquartile range with the line at the median, whiskers extend to 1.5xIQR, points beyond are plotted individually, and n is the number of scenarios in each rating bucket (C) Rating heat maps for BECCS (D) LCCR change compared to benchmark for BECCS (E) Rating heat maps for DAC (F) LCCR change compared to benchmark for DAC. The color reports LCCR scale. See also Figures S5 and S7, and Table S1.

Under merchant pricing, both BECCS and DAC receive an SACP of B-. For BECCS, feasible debt ranges from 14%–27%, well below the 50%–80% percent typical in infrastructure finance and consistent with FOAK lending conventions.^48^^,^^49^ Under full equity financing the cost of removal is US$312 and US$2,620 per tCO_2_ for BECCS and DAC respectively; the DAC figure reflects a fully loaded FOAK facility in the United Kingdom, inclusive of infrastructure electricity prices, contingencies, T&S, and MRV costs; absent these loading factors, costs reduce to US$500–720 per tCO_2,_ consistent with disclosed operating facility costs (see STAR Methods: LCCR verification). Under a stylized 50% leverage, investment-grade rating, scenario, the levelized cost of removal reduces to approximately US$254 and US$2,234 per tCO_2_, but this capital structure is not supported under current merchant conditions. When constrained to B- terms, the levelized cost of removal for BECCS is between US$238 and US$400 per tCO_2_. For DAC, feasible debt is limited to 2%–11%, and the levelized cost of removal ranges from US$2,220 to US$3,500 per tCO_2_.

Under a fixed CDR pricing regime with full coverage for BECCS, the rating improves to B+ with a US$275 per tCO_2_ fixed offtake, and BBB- at US$375 per tCO_2_. These upgrades shift feasible leverage and the resulting cost of removal. At an offtake price of US$375 per tCO_2_, applying BBB- consistent financing terms, the maximum leverage increases to between 32% and 54%, resulting in the levelised cost of removal falling to approximately US$261 per tCO_2_. At US$500 per tCO_2_, the maximum leverage increases to 80% and the cost of removal falls to approximately US$222 per tCO_2_. This represents a reduction of roughly 26 percent relative to the merchant scenario. A similar behavior is also observed with electricity price stabilization. At full coverage and US$275 per MWh, the SACP reaches BBB-, leverage reaches between 34% and 58%, and the levelized cost of removal collapses. While BECCS can achieve an investment-grade SACP within the tested strike price range, a different pattern is observed for DAC. A BBB- rating is only achieved at a fixed price of US$2,750 per tCO_2_, where leverage of between 27% and 47% is enabled and the LCCR is reduced to between US$1,900 to US$3,000 per tCO_2_; roughly 14% reduction compared to merchant.

Partial stabilization yields markedly weaker effects. Under 50% coverage at an offtake price of US$350 per tCO_2_, the levelized cost of removal falls only to between US$223 and US$377 per tCO_2_, with the rating improving marginally to B+ and feasible leverage increasing modestly to between 24% and 40%. Only when coverage reaches 75% and strike prices are at least US$425 per tCO_2_ does BECCS transition toward the investment-grade boundary. These patterns indicate that deep stabilization and sufficiently high strike prices are required to cross the investment-grade boundary. For DAC, partial stabilization does not reach the investment-grade boundary within the tested strike price range.

Input cost stabilization displays a different pattern. With full biomass price stabilization at US$25 per tonne of biomass and no CDR price support, the stand-alone credit profile improves to B+ and feasible leverage rises to between 26% and 42%, but the project remains sub-investment-grade. Over this range, the LCCR falls sharply, from approximately US$312 per tCO_2_ to between US$106 and US$250 per tCO_2_, a reduction averaging 40% driven by lower and less volatile input costs alongside modestly improved financing terms. Even at higher biomass procurement costs of US$75 to US$100 per tonne, full stabilization lowers the LCCR by an average of 27% and 16%, with the stand-alone credit profile remaining sub-investment-grade and in some cases reverting to B-. The overarching observation is that cost containment does not render DAC bankable and that, given its capital intensity, operational cost stabilization alone is insufficient to substantially reduce removal cost.

### Robustness to capital reductions and learning effects

Capital expenditure reductions are used as the primary proxy for learning effects, consistent with the evidence that capital constitutes the dominant cost channel for BECCS and DAC, and that learning rates for capital-intensive technologies substantially exceed those for operating costs. The operating cost dimension is addressed separately through the cost stabilization portfolio analysis.

Reducing BECCS capital expenditures from the base case of US$6,900 per kW by 25%lowers the LCCR by approximately US$75 per tCO_2_ across the tested scenarios. However, the relevant criterion for capital deployment is bankability, and the effect of capital reductions is limited by revenue contractability. In scenarios with stabilized revenues across the tested coverage levels, Figure 5 shows that capital reductions can then materially increase bankability, even when the implied SACP ratings do not change relative to the base case. Under merchant exposure, bankability remains effectively zero despite the cost reduction, because downside cash flow tails from immature markets continue to drive DSCR failures and prevent any meaningful expansion of debt capacity. This robustness test therefore shows that technology learning is complementary to revenue stabilization rather than a substitute for it, since only stabilized revenues allow cost reductions to translate into improved credit quality, lower financing costs, and scalable leverage. We further tested operational cost stabilization portfolios for BECCS. Although these portfolios improve bankability more than isolated cost reductions, their effect remains weaker than revenue stabilization. For BECCS, these results can be distilled into four representative cases, shown in the *graphical abstract*. Under merchant conditions the LCCR is US$312 per tCO_2_ with bankability near zero. An illustrative 40% reduction in capital cost lowers the LCCR by 38% to US$194 yet bankability remains negligible. Creditworthy offtake sufficient to reach investment grade raises bankability to 43% at US$261, and combining the two yields the lowest LCCR (US$161) and the highest bankability (57%).

**Figure 5:**
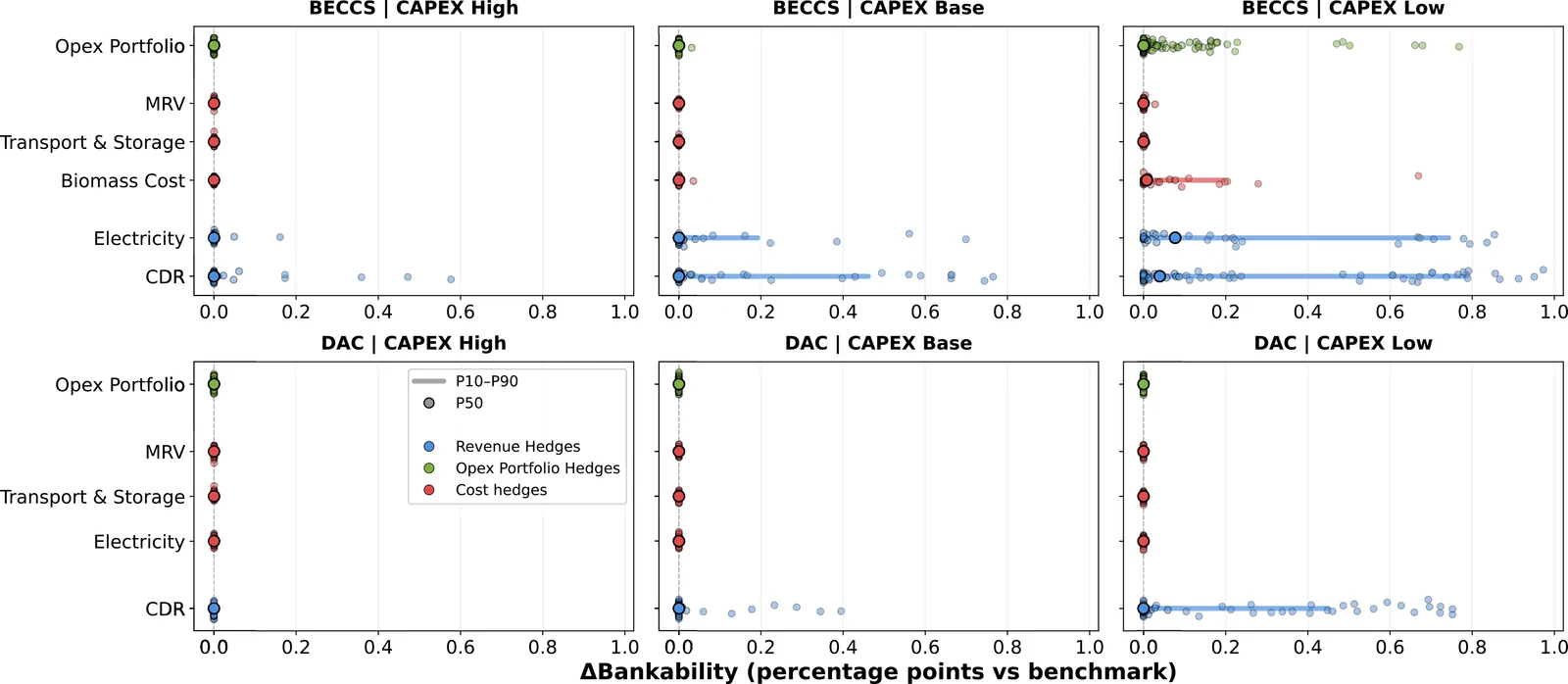
Effect of policy and risk-transfer instruments on bankability Panels show the change in bankability across a variety of scenarios. Each marker corresponds to one scenario, defined by the share of output covered (25%–100%) and the strike price and is plotted for three capital cost assumptions. For each intervention the dot marks the median (P50) and the bar spans the 10^th^ and 90^th^ percentile (P10-P90) of the change in bankability across the scenarios in that group. See also Figures S4, S6, S8, and S9.

An equivalent test for DAC yields the same qualitative conclusion. Capital reductions lower the levelized cost of removal substantially, to between US$1,700 and US$2,750 per tCO_2_, but bankability remains largely unchanged across most scenarios. Only under the highest revenue stabilization settings does bankability improve meaningfully. Together, these results reinforce that revenue stabilization is the binding lever for bankability, while cost reductions primarily reduce the levelized cost of removal conditional on financing being available. The central findings are further robust to assumptions on cost of equity and hurdle rate, operational risk calibration, and counterfactual credit profile (see Methods S1).

## Discussion

To date, most CDR financing has come from public financing programmes or high-risk private equity. Neither source can support the multi-billion-dollar annual capital flows required for durable industry growth. Achieving scale will therefore depend on the entry of institutional capital. Institutional capital—whether from debt markets, pension funds, or sovereign wealth funds—typically only enters once projects offer investment-grade credit profiles, transparently priced returns and long-term contracted revenues.^50^ These conditions have rarely, if ever, been met by CDR projects to date. The gap between what CDR costs in principle and what it costs to build and finance at scale is a central barrier to deployment—a distinction that techno-economic assessments, by treating financing as an assumption rather than an outcome of project risk, have not been able to close. Deterministic discount rates obscure the conditions lenders use to set terms, resulting in a literature that can determine when CDR is competitive in expectation, but not when it becomes bankable in practice. Our results speak directly to that question by showing that policy instruments which stabilize revenues and create long-term contractable cash flows are more effective at unlocking private capital than technology cost reduction programs alone.

### When can CDR enter investment-grade territory?

Our results indicate that stable, contractable revenues appear to be a necessary condition to transition CDR into an investment-grade asset class. Policy-backed mechanisms such as the UK GGR Business Model can manufacture these conditions, but the reliance on a public funding base is likely to constrain long-run scalability. Recent UK draft commercial principles on the Transition Access Agreement signals an expectation that the market should increasingly connect under commercial arrangements rather than rely on open-ended support, reinforcing the view that early public de-risking must give way to market maturation.^51^ Nonetheless, policy mechanisms remain essential in early market formation, because they provide the credibility and cash flow standardization needed to seed market confidence and mobilize FOAK capital. A durable transition therefore requires policy sequencing that is complementary and supportive to the development of robust market-driven pricing and a higher willingness-to-pay for high integrity removals. This is also contingent on how emerging regulatory frameworks, including the EU CRCF, define, differentiate, certify, and reward quality, since these design choices shape what can be contracted and what can be financed.^52^^,^^53^

Investment-grade conditions are theoretically attainable under hybrid revenue structures. This becomes feasible when electricity is sold under long-term PPAs while carbon revenues remain merchant exposed. For BECCS, we find that consumer WTP for firm low-carbon electricity would need to support strike prices in the order of US$250 to US$300 per MWh, which is the range in which the implied credit profile approaches the BBB- investment-grade boundary. This range is closer to early European renewable support levels than to prevailing wholesale electricity prices,^54^^,^^55^ implying that investment-grade outcomes would require long-term bilateral contracts with counterparties willing to pay a premium for firm, low-carbon electricity.

An electricity-anchored revenue model can also accelerate the conversion of capital into contractable cash flows. Removal credits require certification and verification that extend well beyond issuance, which elevates perceived risk and weakens debt capacity.^56^ Electricity sales, by contrast, can generate familiar, near-term, contractable revenues that are more readily underwritten, improving credit quality. In our results, FOAK pathways relying solely on removal credit revenues face a structural commercial constraint, with investment-grade profiles requiring carbon prices above most of those seen in disclosed transactions, even under substantial capital learning. Under these conditions, the existence of willing offtake, rather than expected cost reduction alone, governs the transition to investment-grade financing.

### Do credit conditions dominate technology cost in determining CDR viability?

The dominance of financing cost over technology cost in determining removal cost runs through a single channel: credit quality sets feasible leverage and debt pricing, and these financing conditions are determined by the downside cash flow distribution, not by expected costs. Improvements in credit quality also shift bankability more than comparable capital and operating cost reductions. Moving from sub-investment-grade to investment-grade financing reduces the WACC from 10%–12%to 6%–8%, which lowers the LCCR by approximately 26% for BECCS and 14% for DAC. In comparison, a 25% capital expenditure reduction yields only modest bankability improvements unless revenues are already stabilized, particularly for DAC where high strike prices remain necessary to shift credit ratings.

This arises because credit quality determines feasible leverage, tenor, and debt pricing. Under merchant exposure, BECCS remains in a high cost of capital, low leverage regime in both capital expenditure cases, suppressing bankability even when expected costs fall. Only when revenues are secured through long-term fixed offtake does credit quality improve and financing terms shift sufficiently for bankability to approach investment-grade territory. In this sense, capital learning acts as a complement to revenue stabilization rather than a substitute for it. This sequencing is further illustrated by the counterfactual sensitivity analysis. Extending debt tenor to match an AAA credit profile, absent the revenue predictability required to service it, reduces bankability relative to the base case, whereas holding tenor fixed and applying only the lower all-in rate and higher leverage improve it. The binding constraint is not access to leverage, but the revenue stability needed to justify the debt structure that leverage implies.

### Is bankability a binding constraint on CDR deployment?

Our results show that, for the capital-intensive pathways examined here, bankability behaves as a binding condition for scale because it determines whether projects can access and support investment-grade financing terms. A project can be technically viable and appear cost competitive in expected levelized cost yet remain non-deployable because its cash flow distribution does not support investment-grade credit. When revenues and key costs remain exposed to market uncertainty, downside tails drive covenant breaches, which results in constrained leverage, and debt pricing of a magnitude close to that of equity.

Bankability therefore shapes both the pace of deployment and cumulative removals by determining whether projects can mobilize long tenor, low-cost debt at scale. In our results, financing terms shift discontinuously as projects approach the BBB- investment-grade boundary, and crossing this boundary depends primarily on revenue contractability and downside protection rather than on cost reduction alone. Costs have a more meaningful effect once revenues are contractable. Durable CDR markets must manufacture credit quality through contract structures and shared de-risking that stabilize revenues and protect the downside scenarios. When revenues are contractable, cost reductions translate into lower credit risk, lower financing costs and lower effective removal costs, reinforcing scale through increased debt capacity and higher leverage. When revenues are not contractable, CDR remains reliant on public procurement and high-risk equity, which is insufficient for sustained market growth and meaningful climate impact.

These conclusions apply most directly to capital-intensive CDR pathways with long-lived infrastructure and geological storage obligations, for which project finance is the structurally appropriate financing mode. Pathways with lower capital intensity, shorter payback periods, or distinct contracting conventions—such as enhanced weathering, biochar, and other nature-based solutions—may have different bankability drivers and should not be assumed to share the same revenue-contractability constraint without case-by-case application of the framework. Applying the framework to these pathways requires re-parameterization rather than redesign, replacing the cost, performance, and risk inputs used for BECCS and DAC with pathway-specific values. Debt and project finance are beginning to reach these pathways, including recent enhanced weathering facilities,^6^^,^^57^ so the framework is increasingly applicable to them; their smaller and more distributed scale, revenues that lag upfront outlay, and greater MRV, permanence and delivery risk would, however, reshape the binding constraint. Revenue contractability would remain a constraint, but for enhanced weathering in particular, the cost and uncertainty of MRV would also limit bankability by casting doubt on delivered volumes and the cash flows contracted against them.

The model assumes operational project cash flows are not exposed to construction-phase risk beyond cost escalation. Construction-phase risk would affect bankability mainly through delay and the cost of financing the build. If construction is delayed, revenues are delayed while borrowed capital sits idle; interest then accumulates and is added to the loan, so the plant opens carrying more debt. This results in thinner margins to pay lenders, the main driver of the credit rating, which lowers the rating, reduces the debt that can be borrowed, and raises the cost of removal. Borrowing during construction, and the guarantees lenders require against the risk of non-completion, also cost more than ordinary project debt. Each of these pushes bankability downwards, most severely for FOAK plants, so our results, which assess a project once it is already operating, are best considered as an optimistic case. The same contracted revenues that establish bankability also make construction itself easier to finance, since lenders extend build-phase funding more readily against a secured future cash flow.

Our results carry a direct policy implication: the design priority for early CDR markets is creating contractable, long-duration structures rather than accelerating cost reduction through technology learning alone. The UK Greenhouse Gas Business Model, providing carbon contracts for difference backed by public procurement, and 45Q under the Inflation Reduction Act (IRA) represent precisely the mechanism our framework identifies as necessary to move BECCS into investment-grade territory. For DAC, the required offtake prices remain above most current voluntary market levels, underscoring the continued need for public or blended-finance structures. Recent UK policy signals, including the Transition Access Agreement, indicate an expectation that CDR should progressively stand alone on commercial terms. While specific mechanisms differ by jurisdiction, the sequencing challenge—ensuring commercial markets are sufficiently deep before public support is withdrawn—applies equally to analogous frameworks in the EU, United States, and elsewhere. Our results suggest this transition is viable only if a durable commercial offtake market develops before public support is withdrawn; without it, projects risk returning to merchant exposure and near-zero bankability. The central challenge for CDR market design is therefore sequencing that withdrawal, ensuring commercial revenue structures are sufficiently deep to sustain investment-grade conditions before public backstops are removed.

### Limitations of the study

While this analysis demonstrates that revenue contractability is the primary bankability determinant for BECCS and DAC, several limitations apply. Cost components for both BECCS and DAC are derived from academic literature rather than disclosed project accounts; the limited public project-level data for CDR makes independent validation of assumptions difficult. While DAC costs are calibrated to a fully loaded FOAK facility and sit at the higher end of published estimates, the qualitative bankability conclusions, that DAC requires contractable revenue well above most existing voluntary transactions, hold across a range of cost assumptions, as demonstrated in the capital cost sensitivity analysis. The analysis is further restricted to capital-intensive CDR pathways financed through project finance structures in developed capital markets. The cost of capital for CDR projects remains an active area of research; extending this framework to incorporate empirically validated, project-level cost of capital estimates as they become available would further strengthen the quantitative conclusions.

## Resource availability

### Lead contact

Further information and requests for resources should be directed to and will be fulfilled by the lead contact, Julian Henry Smart (julian.smart21@imperial.ac.uk).

### Materials availability

This study did not generate new unique reagents.

### Data and code availability

All original code for the project-finance and credit-rating model has been deposited at Zenodo and is publicly available as of the date of publication. The DOI is listed in the key resources table. All data supporting the findings are provided in the manuscript and supplemental information. Any additional information required is available from the lead contact upon request.

## Acknowledgments

The authors thank the United Kingdom Research and Innovation (UKRI) for funding this work under the Natural Environment Research Council Science and Solutions for a Changing Planet Doctoral Training Partnership (studentship ref. 2892606) and the Grantham Institute Imperial for financial support for this work. The opinions expressed and arguments employed herein do not necessarily reflect the official views of the UK Government nor the Grantham Institute.

## Author contributions

Conceptualization, J.H.S.; investigation, J.H.S. and N.M.D.; writing – original draft, J.H.S.; writing – review and editing, J.H.S. and N.M.D.; funding acquisition, J.H.S.; supervision, N.M.D.

## Declaration of interests

The authors declare no competing interests.

## Declaration of generative AI and AI-assisted technologies in the writing process

During the preparation of this work the authors used Claude (Anthropic) and ChatGPT (OpenAI) in order to review and provide editorial feedback on manuscript drafts. After using this tool/service, the authors reviewed and edited the content as needed and take full responsibility for the content of the published article.

## STAR★Methods

### Key resources table

**Table:** 

REAGENT or RESOURCE	SOURCE	IDENTIFIER
**Deposited data**		

Model input parameters and uncertainty distributions	This paper (supplemental information)	Tables S2–S5
Credit Spread data (ICE BofA option-adjusted spreads)	Federal Reserve Bank of St. Louis	https://fred.stlouisfed.org/categories/32348

**Software and algorithms**		

CDR project-finance and credit-rating model	This paper	https://doi.org/10.5281/zenodo.21916904

### Method details

We define a framework that estimates how project level uncertainty affects bankability and financing capacity under project finance. The framework quantifies the extent to which risk transfer mechanisms can shift projects from unbankable to bankable and enables identification of the interventions required to create bankable market conditions for CDR; the workflow, from uncertainty sampling through cash flow simulation and credit assessment to financing terms and LCCR, is shown schematically in Figure 1. We consider this from a project financing perspective in which a special purpose vehicle (SPV) is established as a separate legal entity from the sponsor to host the project.

The model developed for this work is founded on a traditional project finance discounted cash flow (DCF) framework that we extend in two complementary dimensions: credit risk analysis and bankability assessment.

First, a DCF model integrates Monte Carlo simulations of technical, cost, and market uncertainties to estimate project bankability. We define bankability as the probability that a project satisfies the minimum debt service coverage ratio requirements and an equity internal rate of return hurdle under a given policy and contracting structure. Second, credit risk is evaluated using an adapted form of the S&P Global Ratings Project Finance Methodology. We develop BECCS and DAC specific criteria for operational stability (ACOS), project specific performance adjustments, and resource and market risk factors.^58^^,^^59^ This allows each pathway to be benchmarked against established credit rating logic and mapped to corresponding financing spreads.

Together, these two layers quantify how credit quality and de-risking interventions influence the levelised cost of carbon removal (LCCR) and financial viability of CDR projects.

#### Cash flow modeling

A discounted cash flow model with Monte Carlo uncertainty forms the foundation of the analysis. As mentioned in the main text, technoeconomic analyses (TEAs) are essential in early-stage technology assessment because they establish prospective cost drivers and levelised costs. However, they typically abstract from the financing frictions that determine whether capacity can be developed and scaled. To account for these frictions under uncertainty, we first evaluate each project under fully market exposed conditions and then phase in risk allocation measures to isolate their incremental effects on cash flow performance.

The model constructs multiple cash flow measures to reflect the economic claims of different capital providers. Free cash flow provides a project-wide measure of economic performance and is commonly used in corporate finance. Debt investors are evaluated on cash flows available for debt service (CFADS), while equity investors receive the residual free cash flows to equity (FCFE) after operating costs and debt service.

(Equation 1) $${FCF}_{i,t}^{C}=\left({EBITDA}_{i,t}^{C}-{Depreciation}_{i,t}^{C}\right)\left(1-\tau \right)+{Depreciation}_{i,t}^{C}-{\Delta NWC}_{i,t}^{C}-{CAPEX}_{i,t}^{C}-{DEVEX}_{i,t}^{C}$$

(Equation 2) $${EBITDA}_{i,t}^{C}={CDR}_{i,t}^{C}+{CB}_{i,t}^{C}-{OPEX}_{i,t}^{C}$$

Here, the ${FCF}_{i,t}^{C}$ denotes the pre-financing free cash flow for project, *C*, at time, *t*, and simulation, *i*. ${CDR}_{i,t}^{C}$ is revenue from the sale of certified removal units generated by technology, *C*, at time, *t*, in simulation, *i*. ${CB}_{i,t}^{C}$ captures revenues from co-benefits, such as electricity sales in BECCS. *τ* is the corporate tax rate.

Although Equation 1 and Equation 2 define project free cash flow, project finance requires cash flow measures aligned with lender and equity claims. We therefore distinguish between free cash flow, cash flow available for debt service, and free cash flow to equity. This ensures consistency with project finance practice, in which debt sizing and credit metrics are based on CFADS, while equity returns are computed on FCFE. For simplicity, we abstract from interest deductibility. In practice, interest deductibility provides a tax shield that would modestly strengthen DSCR and FCFE, but it would not alter the qualitative investment-grade thresholds identified.

(Equation 3) $${CFADS}_{i,t}^{C}={{EBITDA}_{i,t}^{C}-\Delta NWC}_{i,t}^{C}-\tau_{i,t}^{C}$$

(Equation 4) $${FCFE}_{i,t}^{C}={CFADS}_{i,t}^{C}-{DS}_{i,t}^{C}$$

Here, ${CFADS}_{i,t}^{C}$ denotes the cash flow available for debt service, and ${FCFE}_{i,t}^{C}$ denotes the free cash flow to equity. ${DS}_{i,t}^{C}$ is debt service in period, *t*, for project, *C*, in simulation, *i*. $\tau_{i,t}^{C}$ is the tax paid in the respective simulation and period. Free cash flow to equity is the residual cash flow after debt service and is used to evaluate equity investability through the equity internal rate of return.

#### Bankability assessment

For this paper, we define bankability as the probability that a project satisfies predetermined debt and equity hurdles across Monte Carlo simulations. Scenarios are used to evaluate the efficacy of alternative support mechanisms. For robustness, we also report the probability of passing debt and equity hurdles constraints separately, but we define CDR bankability as the joint probability that both constraints are satisfied.

#### Project-finance appraisal metrics

The project is appraised from a lender perspective using coverage metrics, primarily the debt service coverage ratio (DSCR). DSCR is calculated annually given the assumed debt tenor and amortisation schedule. DSCR measures the ability of operating cash flows to meet scheduled debt service. A DSCR of one indicates parity between cash flow and debt service, values below one implies a shortfall, and values above one indicates coverage. In practice, lenders impose minimum DSCR covenants that commonly range from 1.20x or 1.40x or higher depending on the perceived risk.^49^^,^^60^^,^^61^ As a complementary metric, the Loan Life Coverage Ratio (LLCR), as in Equation 6, can be used, however our primary metric adopted is DSCR.

(Equation 5) $${DSCR}_{i,t}^{C}=\frac{{CFADS}_{i,t}^{C}}{{DS}_{i,}^{C}}$$

(Equation 6) $${LLCR}_{i}^{C}=\frac{\sum_{y=x}^{Y}\frac{{CFADS}_{i,y}^{C}}{{\left(1+r\right)}^{y}}}{{Debt}_{i,x}^{C}}$$

Here, *x* denotes the first year in which scheduled debt amortisation begins, and *y* indexes each subsequent year until loan maturity. The loan tenor is therefore distinct from the overall project lifetime, typically beginning after the first operating year and ending before decommissioning.

To account for volatility in operating cash flows, the model can support a debt service reserve account (DSRA) sized as a predefined fraction of the scheduled debt service, funded from earlier cash flows. The reserve is drawn when CFADS is insufficient to meet scheduled debt service and replenished when cash flows exceed the covenant threshold.

(Equation 7) $${DSRA}_{i,t}=\frac{m}{\gamma }\;{DS}_{i,t}^{C}$$

Here, *m* is the number of months of debt service covered by the reserve, and *γ* is the number of months in the reference period, for example 12 months for annual debt service. The DSRA therefore holds ($\frac{m}{\gamma }$) of the scheduled debt service.

Equity investability is evaluated using the levered equity IRR. Equity IRR is computed on free cash flow to equity after debt service and is compared to a hurdle rate representing the required return on equity. Equity IRR differs from IRR as it is computed post financing.

(Equation 8) $$NPV=0=\sum_{t=0}^{T}\frac{{FCFE}_{i,t}^{C}}{{\left(1+IRR\right)}^{t}}$$

#### Project-finance bankability assessment

In project finance appraisal, lenders and equity investors require that minimum hurdle rates are met for financing to proceed. (Equation 9), (Equation 10), (Equation 11), (Equation 12), (Equation 13) formalise how bankability is computed in our framework by linking lender coverage requirements, defined by the minimum DSCR observed over the debt tenor in each simulation, and equity internal rate of return.

(Equation 9) $$\min_{t\in T_{debt}}\left({DSCR}_{i,t}\right)\geq {DSCR}_{crit}$$

Here, *DSCR*_*crit*_ is the minimum covenant requirement and is commonly in the range of 1.20x to 1.40x for energy infrastructure, with higher thresholds often applied to first-of-a-kind and volatile projects.^49^^,^^60^^,^^61^ $T_{debt}$ denotes the set of periods in which scheduled debt service occurs, corresponding to the debt tenor.

(Equation 10) $${IRR}_{equity,i}\geq {IRR}_{crit}$$

Here, *IRR*_*crit*_ denotes the minimum return required by equity investors. Reported hurdle rates vary across investor classes, with empirical studies suggesting 25 to 30 percent and above for early-stage venture capital,^62^ 10 to 15 percent for infrastructure private equity,^46^ and around 6 to 8 percent for mature, core infrastructure held by pension fund investors.^47^ The increased required return is to compensate for greater perceived failure risk.

In this framework, a project is lender feasible when it satisfies the debt coverage condition and equity feasible when it satisfies the equity return condition. We define full bankability as satisfying both conditions simultaneously.

(Equation 11) $$P\left(B=1\right)=P\left(\min_{t\in T_{debt}}\left({DSCR}_{i,t}\right)\geq {DSCR}_{crit}\cap {IRR}_{equity,i}\geq {IRR}_{crit}\right)$$

Equation 11 defines the probability that a given business model is bankable, measured as the share of Monte Carlo simulations in which both the debt and equity conditions are satisfied. Policy and contracting mechanisms are represented as changes in the stochastic cash flows, allowing decision makers to estimate how alternative instruments affect bankability.

While Equation 11 reports bankability as a frequency across simulations, lenders also assess downside performance. We therefore also report tenth percentile outcomes for the minimum DSCR and equity internal rate of return distributions.

(Equation 12) $$P10\left\{minDSCR\right\}=Q_{0.1}\left({\left\{\min\;{DSCR}_{i}\right\}}_{i=1}^{N}\right)$$

(Equation 13) $$P10\left\{IRR\right\}=Q_{0.1}\left({\left\{IRR\right\}}_{i=1}^{N}\right)$$

Reporting downside tails distinguishes cases where only one condition is met. For example, scenarios with high tenth percentile minimum DSCR but low joint bankability indicate structures that are lender feasible, but equity constrained. This is important as maximum leverage is modeled using the P10 of the DSCR distribution given debt is provided contingent on downside robustness.

#### Relation to VaR and CVaR approaches

Our bankability assessment shares the underlying logic of value-at-risk (VaR) and conditional value-at-risk (CVaR), the downside risk measures used in financial risk management.^25^^,^^26^ VaR reports the loss that will not be exceeded at a given confidence level. In our framework, the tenth percentile of the minimum DSCR distribution used to size debt plays the same role, applied to lender coverage rather than to a monetary loss. This reflects how the rating methodology assess coverage, comparing a base case with a downside case that we present using the P50 and the P10 of the minimum DSCR distribution. The closest antecedent in project appraisal is the NPV-at-risk method, which evaluates investment on a confidence-level net present value.^63^ Recent applications in carbon removal place these measures inside the objective function of an energy system optimisation, applying them to the stochastic cost of purchasing power to cover unmet demand alongside deterministic investment and operating costs, so the quantity solved for is how much capacity is deployed across the system rather than the financing of an individual project.^33^^,^^34^ There the measure is evaluated under an assumed normal distribution, adopted to simplify its derivation and noted as case-specific,^34^ whereas our tenth percentile is read from the simulated distribution of the minimum DSCR.

The approaches differ in what they capture. CVaR measures the severity of outcomes beyond the confidence threshold and is well suited to optimising allocation across projects. Our framework instead maps downside outcomes into the credit rating and, through it, the spread, tenor, and leverage a project can obtain, such that financing terms are an outcome of project risk rather than an assumption. The Carbon at Risk measure quantifies downside in delivered volumes, which we hold deterministic. Liability for reversal in geological storage is divided among buffer deductions at credit issuance, the transport and storage operator during operations, and ultimately the state, and its allocation within carbon credit contracts remains too unsettled to assign to project cash flows. The corresponding limitations of our formulation are that the severity of outcomes beyond the tenth percentile enters the assessment only through the resilience and liquidity modifiers, and that we assess individual projects rather than optimising allocation across a portfolio of technologies.

#### Carbon dioxide removal parameters

While this paper uses BECCS and DAC as a case study, the same framework can be applied to analyze other CDR methods. BECCS costs are derived using a bottom-up engineering calculation, while DAC costs are calibrated using parameter analogues from the academic literature.^11^

#### Bioenergy with carbon capture and storage

This BECCS model solves for operating quantities and cost drivers conditional on a specified system size, defined as tonnes of net CO_2_ removed per year. Given a target net removal, we determine the gross CO_2_ that must be captured as a function of capture efficiency, and the corresponding biomass requirement. Although we do not distinguish between biomass types in this paper, these relationships provide the basis for feedstock specific extensions.

(Equation 14) $${{CO}_{2,gross}}_{i,t}=\frac{{{CO}_{2,net}}_{i,t}}{\eta_{C}}$$

(Equation 15) $${Biomass}_{i,t}=\frac{{{CO}_{2,gross}}_{i,t}}{{CI}_{Biomass}}$$

Here, *η*_*C*_, refers to the capture efficiency of the BECCS facility. *CI*_*Biomass*_, is the amount of CO_2_ stored per tonne of biomass. ${{CO}_{2,net}}_{i,t}$, is the net CO_2_ removed after accounting for process emissions, and ${{CO}_{2,gross}}_{i,t}$ is corresponding gross captured CO_2_.

Thermal energy input is determined using the lower heating value (LHV) of biomass. Adjusting for the electrical conversion efficiency and the plant capacity factor yields the implied nameplate electrical capacity, which is used as a cost anchor.

(Equation 16) $$E_{th,i}\left[MJ/yr\right]={Biomass}_{i}\left[t_{dry}/yr\right]*{LHV}_{Biomass}\left[MJ/t_{dry}\right]$$

(Equation 17) $$E_{el,gross,i}\left[\frac{MWh}{yr}\right]=E_{th,i}\left[MJ/yr\right]*\eta_{el}*\left(1MWh/3600MJ\right)$$

(Equation 18) $$P_{el,gross,i}\left[MW\right]=\frac{E_{el,gross,i}\left[\frac{MWh}{yr}\right]}{\left(8760*CF\right)}$$

Here, *E*_*th*,*i*_, is annual thermal energy input. *E*_*el*,*gross*,*i*_ is annual gross electrical output, *η*_*el*_ is electrical efficiency, and *P*_*el*,*gross*,*i*_, is the implied nameplate capacity of the plant. Adjusting for parasitic load, *λ*_*par*_, an estimation of the bioelectricity produced by the facility, *E*_*el*,*net*,*i*_, can be determined at an annual resolution.

(Equation 19) $$E_{el,net,i}=E_{el,gross,i}*\left(1-\lambda_{par}\right)$$

The inputs informing this series of calculations are shown in Table S2.

#### Direct air capture

The DAC module follows a cost model calibrated to the approach in Young et al. (2023), other than CDR efficiency which is taken from Chiquier et al., (2022).^64^ The parameters and component costs are in Table S3.

The individual component costs are scaled to the project system size and aggregated to estimate engineering, procurement, and construction (EPC) costs. We include process and project contingencies to represent cost escalation during development and construction. Total project cost is defined as in Equation 20.

(Equation 20) $${TPC}_{i}={EPC}_{i}+{Process}_{i}+{Project}_{i}$$

Total project cost is then used to estimate additional capital items, including spare parts, owner’s costs, capital provisions, and maintenance. We also include initial working provisions for labor, energy, and chemicals based on expected annual operating requirements. Total capital expenditure is defined as in Equation 21.

(Equation 21) $${Capital\;Expenditure}_{i}={TPC}_{i}+{Owner^{\prime }s\;Costs}_{i}+{Spare\;Parts}_{i}+{Capital}_{i}+{Labour}_{i}+{Fuel}_{i}+{Chemicals}_{i}$$

In both BECCS and DAC, capital learning is applied at the underlying technology cost level, before adding contingencies or other provisions for cost escalation during development and construction.

#### LCCR verification

The Levelised Cost of Carbon Removal used for this work have been verified against existing literature. The merchant costs identified for BECCS, around US$300 per tCO_2_ are well aligned with the values found in the literature.^65^^,^^66^^,^^67^^,^^68^ The implied credit ratings are verified using a report by the Cambridge Economic Policy Associates (CEPA) from 2025 whereby they identify a rating for Drax at around BB + under which some price stabilisation is applied.^69^ Our model produces similar results, with modest price stabilisation showing an equivalent rating.

For DAC our costs are on the higher end of those found in literature at around US$2600. It is important to note that this is specifically for a fully costed, risk exposed, small-scale first-of-a-kind solid DAC facility following the dual top-down and bottom-up costing of Young et al., (2023). When their costs, based in 2019, are scaled for inflation using the Chemical Engineering Plant Cost Index (CEPCI) to their 2024 equivalent, costs in the order of those in this paper are achieved. Furthermore, a recent DAC review shows the cost of removal for a facility costed using the same approach in the United States with nuclear power is between US$1500 and US$3000; our costs sit comfortably within this range while using more expensive UK infrastructure electricity prices and MRV costs.^70^ Similarly, a voluntary carbon market purchase agreement between Stripe and Heirloom was agreed at a price of US$2054 per tCO_2_,^45^ within the range of our costs.

Adopting a similar approach to Young et al., when we assume our model has no contingencies (equivalent application to overruns), T&S costs, MRV costs, access to cheap electricity, and no discount rate, we get a net removal cost of US$730 per tCO_2_, and gross removal cost of US$556 per tCO_2_. These costs, as mentioned in Young et al., are consistent with disclosed costs from Climeworks.

#### Stand-alone credit profile

The stand-alone credit profile (SACP) summarises the relative credit risk of the project SPV. Analogous to issuer ratings for corporates or sovereigns by major credit rating agencies, the SACP maps project specific risks into a letter-grade that governs feasible leverage, tenor and pricing. Here, we adapt S&P’s project-finance methodologies to CDR assets and link the resulting SACP directly to the Monte Carlo cash-flow simulations and bankability metrics described above. Following this architecture, the SACP combines quantitative and qualitative assessments of operating risk, market exposure, and financial robustness for the project under evaluation. The implied rating is specific to the market design and uncertainty calibration used here and is employed as a consistent benchmark for comparing financing structures rather than as a claim about any individual asset in operation. The stand-alone credit profiles reported here are our own estimates, obtained by applying a published rating methodology to simulated project cash flows. They are not credit ratings and have not been assigned, reviewed, or endorsed by S&P Global Ratings.

The financial assessment requires a set of reference financing assumptions, reported in Methods S1. In line with terms observed for established investment-grade infrastructure, we calibrate the reference financing package around conditions typical of a BBB- rating. The SACP produced by the model then reflects the implicit credit risk implied by the underlying operating, revenue, and cost assumptions, which may be above or below BBB-. We abstract from refinancing risk and construction-phase rating. The SACP is assessed at the start of the operations and held constant over the debt term.

#### Operations phase business assessment (OPBA)

Assigning an OPBA requires five risk assessments.

1. Performance Risk
2. Regulatory Risk
3. Resource Risk
4. Market Risk
5. Country Risk

Performance risk is captured through the asset class operational stability (ACOS), which reflects the complexity and maturity of the asset and the volatility of its operating cash flows. ACOS scores range from 1 to 12, with higher scores indicating greater operational complexity and uncertainty. We assign ACOS scores to BECCS and DAC based on technology readiness level (TRL), operating track record, and process complexity. Tables S6 and S7 document these assessments for the main CDR pathways, and Table S8 sets out the project specific attributes that would adjust these scores in an individual transaction.

The initial ACOS score is then adjusted for the project’s exposure to regulatory and resource risk. Because CDR is an emerging industry with evolving regulation, we apply a one notch negative adjustment to reflect current policy uncertainty. For sectors with established and widely recognised regulation, the regulatory filter has no effect.

Resource risk captures the likelihood that output is constrained by insufficient resource volume or quality. For BECCS, this primarily reflects sustainable biomass availability, which is limited by geophysical conditions, land use constraints, and social acceptability. Table S9 summarises how we map these constraints into relative resource risk assessments across the two pathways. The ACOS score is adjusted by one to four notches depending on the assigned resource risk category.

Market risk captures exposure to price and volume fluctuations and the extent to which these propagate into operating cash flows. Projects whose CFADS is highly sensitive to market conditions are assessed as riskier than those with stable, contracted revenues.

We quantify market risk using the distribution of CFADS generated by the Monte Carlo simulations. Specifically, we compare the median CFADS to the tenth percentile CFADS and interpret the wedge between these statistics as a measure of downside cash flow volatility. This wedge is then mapped to discrete market risk categories using the market exposure assessment bands set out in the general project finance rating methodology (Table 5, p. 20).^59^

Country risk reflects geopolitical, legal, and macroeconomic stability in the project jurisdiction. In this study, we restrict attention to projects located in high income jurisdictions that we treat as low country risk environments. As a result, country risk does not modify the OPBA in our baseline analysis.

In summary, we assign an initial ACOS score based on performance risk, adjust it for regulatory and resource risk to obtain an intermediate operational risk score, and combine this with the market risk assessment to derive a preliminary OPBA. We then apply the country risk modifier to obtain the final OPBA used in our analysis. The mapping from scores to assessment outcomes follows the preliminary and final OPBA tables of the general project finance rating methodology (Tables 6 and 7, pp. 23 and 24).^59^ Country risk is assessed as low for all jurisdictions, so the final OPBA equals the preliminary OPBA in all scenarios.

#### Operations phase financial assessment (OPFA)

The operations phase financial assessment evaluates whether the project generates sufficient cash flow to meet its financial commitments. This assessment is based on CFADS and DSCR. The model computes DSCR as described previously and uses the minimum DSCR over the debt term as the primary indicator of financial robustness.

The full S&P methodology includes tests for intra year volatility and shortfalls. We abstract from these for tractability given the annual resolution and purpose of our framework. Instead, we incorporate two modifiers, liquidity and resilience. Resilience, defined in Table S10, depends on the frequency with which DSCR falls above or below specified thresholds across simulations. Liquidity, defined in Table S11, is evaluated using the sources-and-uses ratio, the DSRA profile, and CFADS sensitivity to shocks. The liquidity and resilience adjustments are conservative and bounded between +2 and −2.

#### Stand-alone credit profile (SACP) assignment

The OPBA and OPFA are combined to determine the SACP for the project, following the preliminary SACP table of the general project finance rating methodology (Table 8, p. 27).^59^

#### Credit-adjusted levelised cost of removal

The credit risk assigned to a project determines the financing terms it can access and, in turn, its effective cost of carbon removal. In our framework, the SACP governs three parameters, the minimum DSCR requirement, feasible debt tenor, and the credit spread on debt. Together, these parameters determine the maximum feasible leverage and the cost of debt applied in the simulations.

The SACP is mapped to rating consistent financing terms using Table S1 and Figure S1. A BBB- profile, corresponding to the lowest investment-grade category, is associated with more lenient DSCR covenants, based on the rationale that riskier projects require higher coverage,^49^^,^^60^^,^^61^ longer tenors, and lower spreads when compared to a B- profile. Rating linked terms are then used to size the maximum feasible debt and to compute a credit-adjusted LCCR for each project configuration.

#### Maximum feasible debt, cost of debt, and credit-adjusted cost of carbon removal

The SACP determines the minimum DSCR constraint applicable to the project, shown in Table S1. Because CFADS is independent of capital structure, we use the simulated CFADS paths to solve, for each simulation, the maximum level of debt that can be serviced while maintaining DSCR at or above the rating specific minimum in all debt service periods. We also impose an upper bound on leverage of 80 percent, consistent with the upper end commonly observed in project finance. This procedure yields a distribution of maximum feasible debt ratios conditional on the SACP. Equation 22 shows the equation for maximum leverage, whereby *T*_*d*_ is the debt tenor, which is different from *T*, the project life.

(Equation 22) $${L^{*}}_{i}^{C}=\min\left(\frac{\sum_{t=t_{d}}^{T_{d}}\frac{{CFADS}_{t,i}^{C}}{{minDSCR}_{t,i}^{C}}\cdot {\left(1+r_{d}\right)}^{-t}}{\sum_{t=1}^{T}{CAPEX}_{i,t}^{C}\cdot {\left(1+r_{d}\right)}^{-t}},0.8\right)$$

The SACP is also mapped to rating specific credit spreads reported in Figure S1. These spreads are added to a risk-free reference rate to obtain a credit-adjusted cost of debt. For UK based projects, we use the 20-year UK gilt yield as a duration-matched approximation for the risk-free rate. Together, maximum leverage and the cost of debt define the financing structure applied to each scenario and feed into the calculation of the credit-adjusted LCCR.

The credit-adjusted LCCR is computed using the capital recovery factor (CRF), which annualises the total capital expenditure at the rating-implied WACC:

(Equation 23) $${WACC}_{i}^{C}={r_{d}}_{i}^{C}\cdot \left(\frac{D}{D+E}\right)\cdot \tau_{i,t}^{C}+{r_{e}}_{i}^{C}\cdot \left(\frac{E}{D+E}\right)$$

Where ${r_{d}}_{i}^{C}$ is the rating-aligned cost of debt, ${r_{e}}_{i}^{C}$ is the cost of equity, $\tau_{i,t}^{C}$ is the tax rate, *D* is the proportion of debt in the capital structure, and *E* is the proportion of equity.

(Equation 24) $${CRF}_{i}^{C}=\frac{{WACC}_{i}^{C}{\left(1+{WACC}_{i}^{C}\right)}^{n}}{\left[{\left(1+{WACC}_{i}^{C}\right)}^{n}-1\right]}$$

The LCCR is then:

(Equation 25) $${LCCR}_{i}^{C}=\frac{\left({CAPEX}_{i,t}^{C}\cdot CRF\right)+{OPEX}_{i,t}^{C}-{CB}_{i,t}^{C}}{\gamma_{i}}$$

Where ${WACC}_{i}^{C}$ is the project weighted average cost of capital implied by the SACP, *n* is the project life in years, ${CAPEX}_{i,t}^{C}$ is the total capital expenditures, ${OPEX}_{i,t}^{C}$ is the average annual operating cost, ${CB}_{i,t}^{C}$ is the average annual revenue associated with co-benefits e.g., bioelectricity for BECCS, and *γ* is the average annual net removal volume. This decomposition isolates the financing effect (the CRF multiplier on CAPEX), enabling direct attribution of changes in LCCR to credit quality versus technology cost.

### Quantification and statistical analysis

Uncertainty assigned to input parameters is determined by the availability of public data. Although the limited empirical record for CDR is well documented, project level distributions can be constructed using publicly available observations and analogues from adjacent sectors. The distributions are used to run Monte Carlo simulations whereby we run 2,500 simulations per scenario. Having tested 10,000 simulations, we reduced to 2,500 given similarities in distribution results and the computational model efficiency.

For input resources such as electricity and biomass, historical price series provide a basis for estimating distribution parameters. For parameters that are CDR specific, including measurement, reporting and verification (MRV) and T&S, direct observations are sparse. We therefore draw on published technoeconomic analyses and related academic and gray literature to specify triangular distributions using lower bound, central, and upper bound values that are consistent with the cited evidence base. The distributions used in this analysis are reported in Tables S4 and S5, with graphical summaries provided in Figures S2 and S3. Prices and cost inputs are stated as of February 2026.

Where possible, we abstract from independent price forecasting to preserve the focus of the analysis on financing constraints and risk allocation. T&S costs are taken from the cited sources and are calibrated to a representative case of a 100-mile transport distance in a one million tonne per annum pipeline, which reflects a developer connecting to an established T&S network at short distance to geological storage. We do not consider non-pipeline transport.
