# Supplementary material for: Why cost reduction alone cannot deliver bankable carbon dioxide removal
Source: iScience. 2026 Sep 30;29(10):117702. doi: 10.1016/j.isci.2026.117702 (PMC13657177; doi:10.1016/j.isci.2026.117702)
Supplement: Supplementary file 1 [file mmc1.pdf]

iScience, Volume *29*

Supplemental information

Why cost reduction alone cannot deliver bankable carbon dioxide removal

Julian Henry Smart and Niall Mac Dowell

Methods S1: Counterfactual financing structure, credit-rating criteria, and sensitivity analysis

Counterfactual Financing Structure

The counterfactual defines the baseline financing structure against which all rating linked, and policy induced adjustments are evaluated. It reflects the terms a developer could reasonably expect in the absence of formal credit support, guarantees, or an established rating, consistent with the early commercial stage of a CDR project prior to any policy-driven risk transfer.

By holding these parameters constant across all business model configurations, any variation in the resulting credit assessment reflects differences in project fundamentals – revenue structure, risk transfer, and cash flow uncertainty – rather than differences in assumed debt terms. The parameters are intentionally conservative, roughly aligned with the lowest investment-grade rating: in the absence of an established track record or secondary market for CDR assets, we abstract from comfortable terms to remain closer to the terms capital would require at first engagement.

Debt-to-Equity Ratio

We adopt a 50 to 50 debt-to-equity structure as the counterfactual. This is below the 60 to 80 percent leverage commonly observed in contracted infrastructure projects [S1], yet above the 30 to 40 percent often reported for first-of-a-kind or merchant assets [S2]. The midpoint reflects a project with some commercial credibility but without the contracted revenue certainty that unlocks higher gearing, providing a conservative representation of early commercial CDR while preserving comparability with established project finance benchmarks.

Debt Tenor

We assume a ten-year tenor in the counterfactual, consistent with the shorter maturities applied to first-of-a-kind and high-uncertainty technologies relative to conventional project finance [S2]. Lenders shorten tenor to limit exposure to untested operational and offtake risk; this is well-documented in early clean energy demonstrations and represents a structural conservatism appropriate to CDR at its current stage of market development.

Cost of Debt

The cost of debt is defined as the sum of a duration-matched risk-free rate and a credit spread corresponding to a BBB- rated borrower. BBB- is selected because it marks the boundary between investment-grade and sub-investment-grade debt, providing a consistent and well-defined reference point from which rating upgrades and downgrades are evaluated systematically.

DSCR Thresholds

We assess bankability across a range of DSCR thresholds to capture heterogeneity in lender risk appetite across infrastructure sectors. Minimum DSCR covenants span 1.20x, representative of mature, contracted assets, to 1.60x, consistent with first-of-a-kind or commodity exposed projects where lenders require higher coverage buffers [S1], [S2]. This range brackets both established and emerging risk profiles and supports cross-technology comparison under varying debt constraints.

DSRA

The counterfactual assumes a six-month DSRA, consistent with standard project finance practice in which lenders require a liquid reserve to cover near-term debt service obligations [S3]. Cash equivalent to six months of scheduled debt service is prefunded and ring-fenced; excess CFADS above this reserve accrues to equity as residual cash. This reserve is held constant across the counterfactual and serves as the baseline from which the rating-aligned scenarios are evaluated, where the DSRA is sized to the rating-appropriate standard drawn from S&P debt term conventions.

OPBA Selection

The OPBA inputs influence the resulting SACP. Score assignments follow the qualitative descriptors in "*Credit Rating Criteria Adjustment for CDR*" below, with technology-specific calibrations reflecting the relative operational maturity of each assessed pathway. In the BECCS counterfactual, we assume medium operational complexity, with an asset class operational stability score of 6, very low country risk, no standardised regulation, and medium resource risk, scored as 2. For DAC, we assign higher operational complexity, with an asset class operational stability score of 8, reflecting lower technology readiness relative to BECCS. We assume no country risk, no standardised regulation, and low resource

risk, scored as 1. Market risk and all operations-phase financial assessment inputs are endogenous model outputs.

Mapping Ratings to Debt Contract Terms

We report rating specific debt terms calibrated from the literature and market data described in the main text. Longer tenors are assigned to higher credit quality, consistent with observed practice in project and corporate lending. All-in credit spreads are proxied using ICE Bank of America high yield indices for United States issuers, reflecting data availability, and mapped to the relevant rating buckets. Minimum DSCR covenants are not directly reported as a function of rating in the available sources, so we specify DSCR thresholds using the standard underwriting principle that weaker credit quality requires higher coverage to protect against downside stress. The same principle is applied to the sizing of the DSRA across rating categories. Weaker credit ratings are associated with a higher yield spread and lower leverage capacity. Analyses show firms with a credit rating having higher leverage capacity than unrated firms [S7].

Sensitivity Analysis

The following tests assess whether the paper's central conclusions are robust to three key modelling assumptions: the cost of equity and equity hurdle rate, the operational performance baseline assessment (OPBA/ACOS), and the counterfactual credit rating. All tests assume a 100% CDR hedge. Results are consistent across BECCS and DAC.

Cost of equity and equity hurdle rate

The base case assumes a cost of equity and equity IRR hurdle rate of 12%. Under an optimistic 7%, meaningful bankability for BECCS emerges at US$200 per tCO_2 and peaks at approximately 96% by US$450 per tCO_2, before declining at higher offtake prices as the increased revenue enables greater leverage which, under stochastic conditions, increases the probability of covenant breach. Under the base case, bankability remains zero until US$300 per tCO_2 and reaches 76% at US$450 per tCO_2. Under the pessimistic 15%, bankability remains zero until US$350 per tCO_2 and reaches only 53% at US$450 per tCO_2. For DAC, the effect is more pronounced: the optimistic case produces meaningful bankability from US$2000 per tCO_2, reaching 81% by US$3000 per tCO_2, whereas the pessimistic case remains near-zero across the full tested range, reaching only 5.5% at US$3000 per tCO_2. In all cases,

merchant conditions produce zero bankability regardless of equity assumptions, and revenue contractability remains the necessary condition for bankable projects structures.

OPBA and ACOS baseline

The OPBA is the most subjective input in the rating framework, relying on qualitative risk assessments. We test one grade above and one grade below the base case OPBA assessments in “*OPBA Selection*” above, except for country risk, which was held constant, and allow for regulation in optimistic scenarios. For BECCS, the bankability onset price is unchanged across all three cases (US$300 per tCO_2), with the optimistic case producing modestly higher bankability thereafter and the pessimistic case producing modestly lower values. For DAC, differences are similarly small. At US$2750 per tCO_2, optimistic, base, and pessimistic cases produce 12.9%, 12.9% and 7.9% bankability respectively. The OPBA sensitivity produces the smallest effect of the three tests, indicating that the paper’s conclusions are not sensitive to the specific operational risk calibration chosen.

Counterfactual credit rating assumption

The credit risk framework is benchmarked against a counterfactual (see “*Counterfactual Financing Structure*” above). We test the sensitivity of this benchmark to the assumed counterfactual credit profile: BBB-, CCC, and AAA. For both BECCS and DAC, the BBB- and CCC counterfactuals produce near-identical results across the full price range, confirming that the headline findings are insensitive to the assumed baseline in terms aligned to the lower credit range. The AAA counterfactual produces a worse bankability outcome at higher offtake prices. For BECCS, bankability under the AAA counterfactual diverges sharply from US$450 per tCO2 onwards, falling to 43% against a base of 76%. For DAC, the divergence is more modest but directionally consistent. The mechanism is not leverage per se but tenor: the longer debt terms implied by an AAA credit profile extend debt service obligations beyond the horizon that CDR revenue streams can reliably support. Current CDR markets lack the price predictability and contract depth to justify long-term debt structures, such that extending tenor increases exposure to adverse outcomes rather than reducing financing cost. When tenor is held fixed at ten years and only the lower all-in rate and higher leverage of the AAA profile are applied, bankability improves, confirming that the rate and leverage effects are individually beneficial. This finding reinforces the central argument: the binding constraint is contractable, predictable revenue, and in its absence, more aggressive debt structures amplify rather than mitigate financial risk.

Credit Rating Criteria Adjustment for CDR

The architecture follows the general and sector-specific project finance rating methodologies of S&P Global Ratings [S4], [S5], adapted for CDR assets. Departures are stated where they occur. We assign asset class operational stability scores specific to BECCS and DAC, apply one notch regulatory adjustment reflecting the immaturity of CDR regulation, define resource risk against biomass and electricity supply, quantify market risk from the simulated distribution of cash flow available for debt service, and abstract from the intra year volatility and shortfall tests. The thresholds reported below are those implemented in our model. The thresholds and assessments reported in this section, and the stand-alone credit profiles derived from them, are our own. They are not credit ratings and have not been assigned, reviewed, or endorsed by S&P Global Ratings or similar agencies.

Supplementary Tables and Figures

Rating	Tenor	All-in Spread of Risk-free	DSCR	DSRA (months)
AAA	20	48	1.20x	3
AA	15	63	1.20x	3
A	12	90	1.25x	6
BBB	11	136	1.30x	6
BB	9	243	1.35x	9
B	7	389	1.45x	9
CCC	≤5	879	≥1.60x	12

Table S1. Debt contract terms by credit rating, Related to STAR Methods. *Mapped credit ratings to the debt terms [S4]-[S6]. The all-in spread of risk-free rate is reporting the data as of October 21st, 2025. The 5-year averages are 48, 63, 90, 136, 243, 389, and 879 for AAA to CCC respectively (yield curve in Figure S1).*

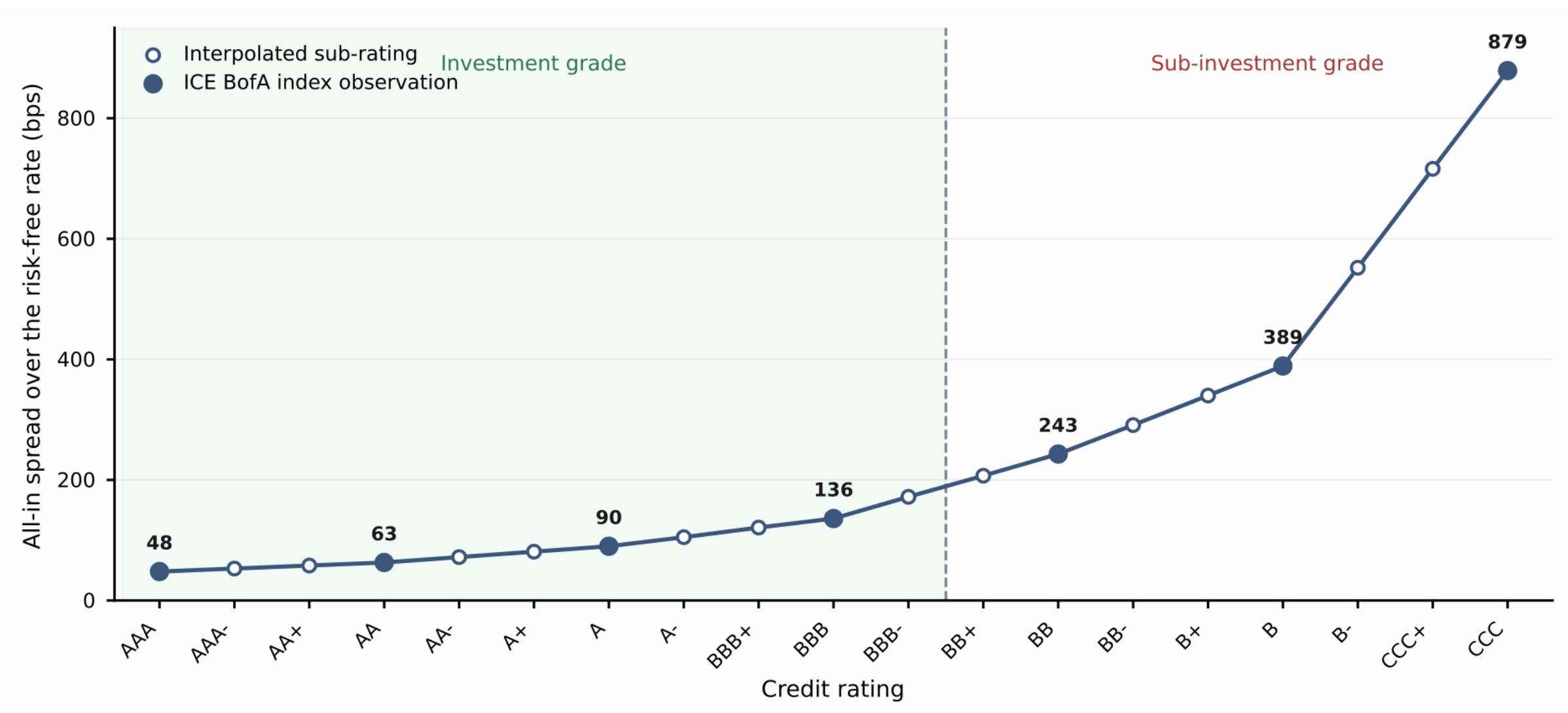

Figure S1. BofA credit spread curve by rating, Related to STAR Methods. *BofA credit spread curve (from FRED). Interpolated ratings (AAA, AA, A etc.,) to get the spread for intermediate ratings (AAA-, AA+, AA- etc.,)*

Parameter	Value	Unit	Source
CO_2 Removed	25,000	tCO_2	Own Assumption
CDR Efficiency	74.68	%	[S8]
Biomass Carbon Intensity (derived from carbon content)	1.69	$\frac{CO2}{t_{biomass}}$	[S9]
MJ-to-kWh conversion factor	0.2778	-	Standard Rate
Electrical Efficiency	40.00	%	Own Assumption
Heating Value Biomass	18.6	MJ/kg	[S10], [S11]
Capacity Factor	85	%	Own Assumption
Parasitic Load	19	%	[S12], [S13]

Table S2. BECCS operational parameters, Related to STAR Methods. *Operational parameters for the BECCS module*

Parameter	Value	Unit
CO_2 Removed	25,000	tCO_2
CDR Efficiency	76.10	%
Electricity Requirement	0.99	GJ per tCO_2
Heat Requirement	9.8	GJ per tCO_2
Sorbent Cost	37	US$ per tCO_2
Owner’s Costs	7	% of TPC
Spare Parts	0.5	% of TPC
Start-up Capital	2	% of TPC
Start-up Labour	25	% of annual direct and indirect labour
Indirect Labour	30	% of direct labour and materials
Maintenance	1.5	% of TPC

Start-up Fuel Energy	2	% of annual fuel/energy
Start-up Chemicals/Minerals	8	% of annual chemicals/minerals
**Solid Sorbent (0.960 ktCO_2 per year)**		
Contractors	1.14	US$M
Blowers	0.668	US$M
Vacuum Pump 1	0.0999	US$M
Vacuum Pump 2	0.0650	US$M
Gas Storage Balloon	0.002	US$M
Condensers	0.242	US$M
Switching Valves	1.19	US$M
Initial Sorbent	0.105	US$M
Buildings	0.166	US$M
Compressor	1.31	US$M
Heat Pump	0.359	US$M
COP	2	-
Operator per mtCO_2 capacity	278	-
Operator Salary	70800	US$ per year

Table S3. DAC operational parameters and component costs, Related to STAR Methods. *Operational parameters for the DAC module. All values below solid sorbent refer to the parameters used to determine the Total Installed Cost. Values shown are in USD 2019. The model inflates the values to USD 2024 using CEPCI*

**Probabilistic Variable**	**Initial Distribution**	**Source**
MRV Costs (%)	Tri(1, 15, 27)	[S14]
T&S Costs (100 miles, 1mtpa [US$ per tCO_2])	Tri(12.6, 23.3, 45.7)	[S15]
Biomass Costs ($ per tonne biomass)	Normal(158, 26.1)	[S16]
Overruns (%)	Normal(12.8, 30.65), truncated at 0	[S17]
Credit Price (US$ per tCO_2)	Tri(80, 220, 300)	[S18], [S19]

Electricity Price (US$ per MWh)	Normal(124.5, 21.8)	[S20]

Table S4. BECCS uncertainty distributions, Related to STAR Methods. *Uncertainty distributions for BECCS*

Probabilistic Variable	Initial Distribution	Source
MRV Costs (%)	Tri(1, 13, 25)	[S14]
T&S Costs (100 miles, 1mtpa [US$ per tCO_2])	Tri(12.6, 23.3, 45.7)	[S15]
Process Contingency (%)	Uniform(5, 20)	[S21]
Project Contingency (%)	Normal(14.9, 44.7), truncated at 0	[S17]
Credit Price (US$ per tCO_2)	Tri(440, 775, 2054)	[S18]-[S23]
Electricity Price (US$ per MWh)	Normal(231.0, 55.0)	[S20], [S24]
EPC (% of TPC)	Normal(15.18, 2.02)	[S17]

Table S5. DAC uncertainty distributions, Related to STAR Methods. *Uncertainty distributions for DAC*

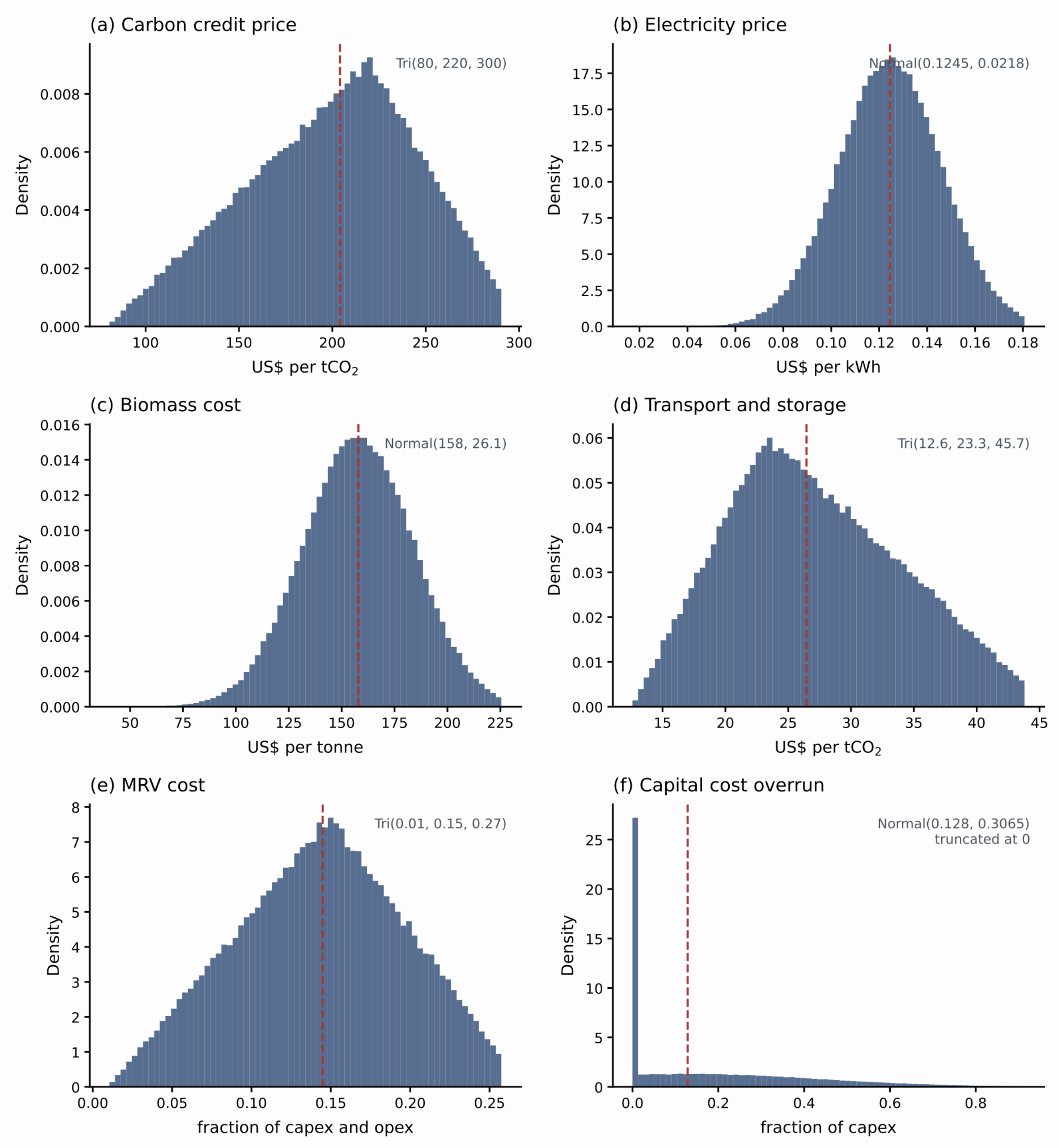

Figure S2. Input distributions for the stochastic BECCS variables, Related to STAR Methods. *Panels (a) is for carbon credit revenue per ton of CO2 (b) electricity revenue per kWh (c) biomass cost per ton of feedstock (d) T&S cost per tCO₂ (e) MRV costs as a percentage of capital and operational expenditures (f) capital expenditure cost escalation as a percentage of capital expenditure*

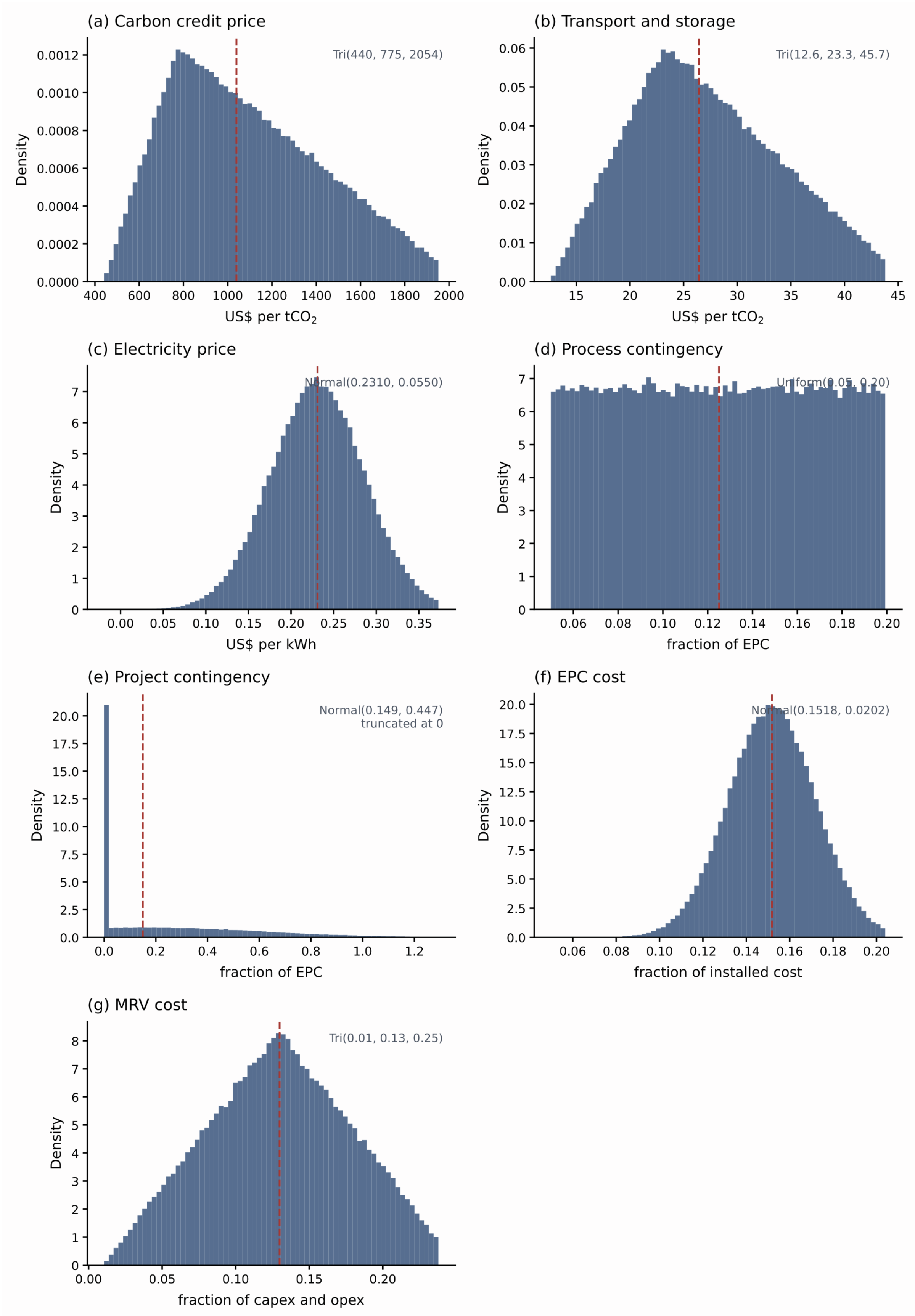

Figure S3. Input distributions for the stochastic DAC variables, Related to STAR Methods. *Panels (a) is for carbon credit revenue per ton of CO_2 (b) T&S cost per tCO_2 (c) industrial non-domestic electricity price per kWh,*

accounting for additional charges (d) process contingency as percentage of total EPC costs (e) project contingency as percentage of total EPC costs (f) EPC costs as a percentage of total installed costs (g) MRV costs as a percentage of capital and operational expenditures

CDR Method	TRL	ACOS (Point + Range)	Rationale	References
DACCS	6	8 (7-8)	Energy-intensive continuous process; sorbent or solvent management; large-scale CO_2 compression; sustained grid or heat dependency. Few commercial-scale operational references exist. Modular configurations co-located with a dedicated heat source and without third-party T&S anchor the lower end.	[S21], [S25]-[S28]
BECCS – Power	7	6 (6-7)	Coupled biomass combustion and post-combustion capture require coordinated scheduling across two operationally distinct systems; biomass feedstock variability introduces flue gas composition uncertainty absent in fossil-fuel CCS analogues. No commercial-scale BECCS-power reference currently exists; the Drax pilot represents the only sub-commercial data point. Configurations with established LTSAs and commercial T&S	[S29]-[S33]

			infrastructure anchor the lower end.	

__Table S6. Asset Class Operational Stability (ACOS) of CDR pathways, Related to STAR Methods.__ ACOS for the methods analysed in this paper based primarily on their respective TRL, the relative complexity of the process, and the likelihood the asset will meet its performance expectations

ACOS Rating	Points	Rationale
Least Complex	1-2	Proven technology, stable operations, predictable cash flows
Low Complexity	3-4	Mature technology, minor operational variations
Medium Complexity	5-6	Established technology, moderate operational complexity
High Complexity	7-8	Advanced technology, significant operational challenges
Most Complex	9-10	Cutting-edge/experimental technology, highly uncertain cash flows

Table S7. General ACOS rating assignment by overall complexity, Related to STAR Methods.

Project-Specific Attribute	Examples of Favourable Adjustments	Examples of Unfavourable Adjustments
Performance Redundancy	Modular DAC arrays (multiple fans/compressors); biochar parks with spare kilns; multiple T&S routes or buffer storage	Single capture train; single compressor; single pipeline/store dependency; no inventory buffer for biomass or sorbent
Operating Leverage	High variable cost share (DAC PPA; flexible biomass contracts); revenue floors that dampen CFADS sensitivity	High fixed O&M and maintenance CAPEX relative to revenue; exposure to spot carbon price and

		merchant power; fixed price feedstock with volume risk
O&M Management	LTSA with performance guarantees; experiences EPC/O&M with CDR references; digital monitoring and planned outages aligned to offtake	New O&M team for FOAK capture; remote sites without service access; unproven marine operations for ocean methods
Technological Performance	Commercial references of the same process & scale; proven solvent/sorbent degradation rates; stable capture efficiency; de-bottlenecked heat integration	Novel chemistries or reactors; scale-up beyond references; unstable capture rates; high heat-rate penalties; corrosion/ash or fouling issues (BECCS/ERW)
Performance Standards (Contractual)	Offtakes, availability-based payments; generous ramp/curtail rules	Stringent guarantees close to theoretical limits; mismatched tenors vs debt
Other Operational Risk Factors	Robust workforce/union stability; benign weather/logistics	Labour constraints; harsh climate access; unexpected major maintenance frequency
MRV Complexity (CDR-specific)	Mature MRV protocol aligned to registry; continuous metering; custody chain with audited book-and-claim; simple boundaries (BECCS post-combustion; DAC at stack)	Novel or spatially diffuse MRV (ocean alkalinity, blue carbon, ERW over large geographies); intermittent sampling; protocol uncertainty that risks volume disallowances

Table S8. Project specific attributed affecting ACOS rating, Related to STAR Methods. *For this framework our results do not consider these adjustments, however, it is crucial to recognize the benefits and drawbacks of project-specific attributes.*

**CDR Method**	**Resource Risk**	**Rationale**	**References**
BECCS-power	Medium	Biomass supply subject to seasonal variability, geographic concentration, and competition from adjacent uses; one dominant feedstock source typical at project level	[S31], [S32], [S34]
DAC	Low/Medium	Grid electricity dependency introduces supply risk under merchant exposure; firm PPA or co-located low-carbon heat source reduces this materially	[S35], [S36]

Table S9. Resource risk assessment categories, Related to STAR Methods. *Assesses the chance that the project could experience a shortfall in production due to a lack of biomass or electricity. Assessment categories defined for this study.*

**Resilience Assessment**	**DSCR Condition**	**Liquidity/OPBA Condition**	**Rating Effect**
**Very High**	Always >= 1.0 under stress	Exception DSCR cushion + strong reserves + OPBA <=6	AA/A: +1; BBB/BB/B: +2
**High**	Always >= 1.0 under stress	Strong reserves or OPBA <= 6	AA/A: 0; BBB +1; BB/B: +2
**Moderate**	Always >= 1.0 under stress	Liquidity sufficient >= 5 years	AA/A: cap at BBB; BBB: 0; BB: +1; B: +2
**Modest**	Always >= 1.0 under stress	Liquidity sufficient >= 3 years	AA/A/BBB: cap at BB; BB: +0; B +1

**Low**	DSCR fails >= 1.0x at any point	-	All categories capped at B

Table S10. Resilience adjustments, Related to STAR Methods. *Resilience adjustments as implemented in this study. Resilience levels are assigned using the criteria in this table, which differ from those of the published methodology; the notching consequences follow it.*

**Liquidity Level**	**Performance Criteria**	**Rating Effect**
**Inadequate**	Any of: (1) 12-month forward coverage < 1.0x; (2) DSRA <6 months debt service; (3) Minimum DSCR < 1.05x; (4) Covenant headroom breached under 10% (OPBA 1-4) or 15% (5-12) CFADS stress	-1
**Adequate**	All inadequate triggers avoided; strong liquidity not fully met	0
**Strong**	OPBA 1-6, sources and uses > 2.0x + minimum sustained reserves >= 12 months; OPBA 7-12, sources and uses > 2.5x +18-months; ample covenant headroom; distribution tests satisfied throughout tenor	+1

Table S11. Liquidity adjustments, Related to STAR Methods. *Liquidity adjustments as implemented in this study. Liquidity levels are assigned using the criteria in this table; the conditions condense the criteria of the published methodology and apply our own thresholds.*

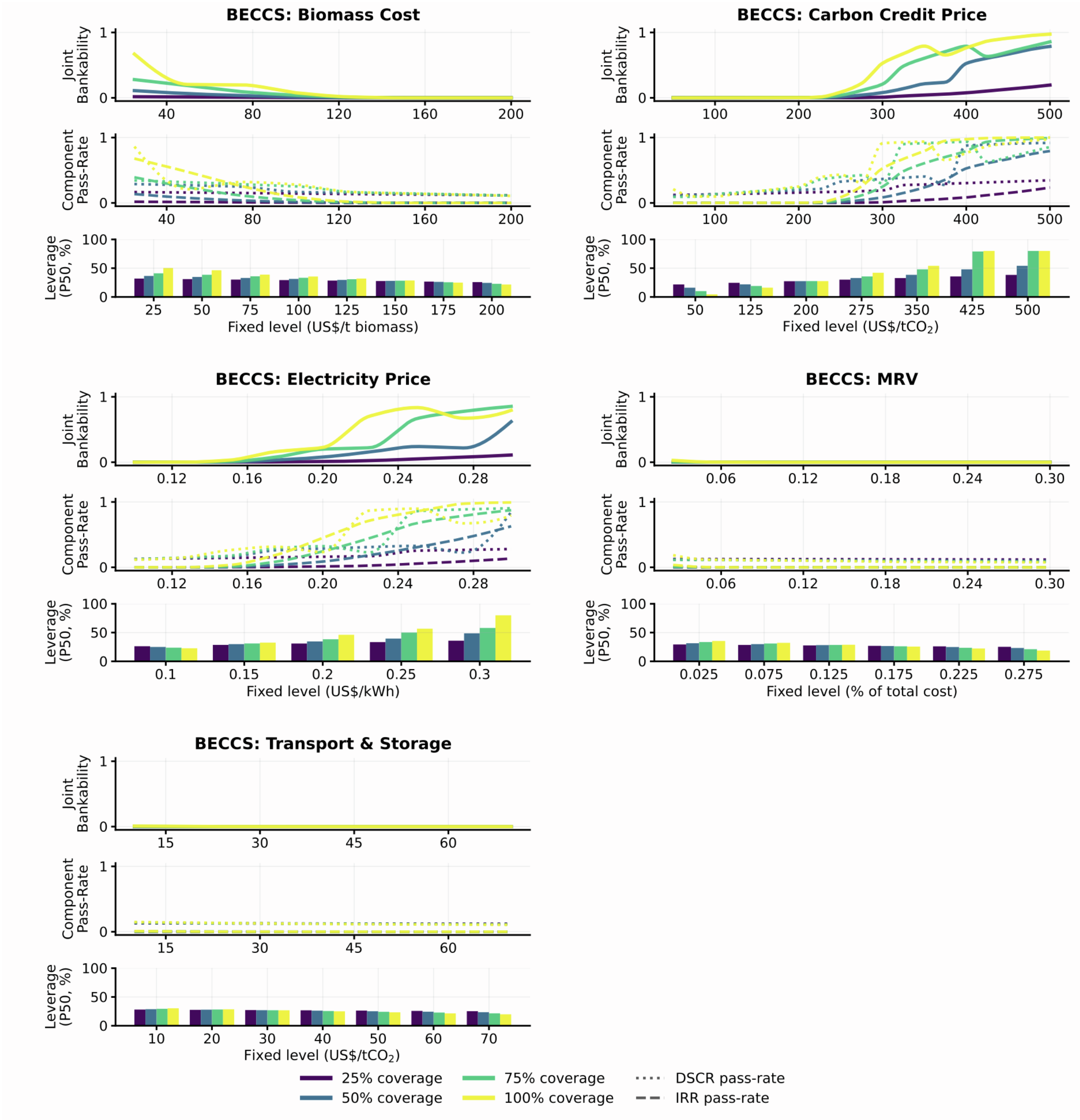

Figure S4. Bankability curves for BECCS under low capital cost scenario, Related to Figure 3.

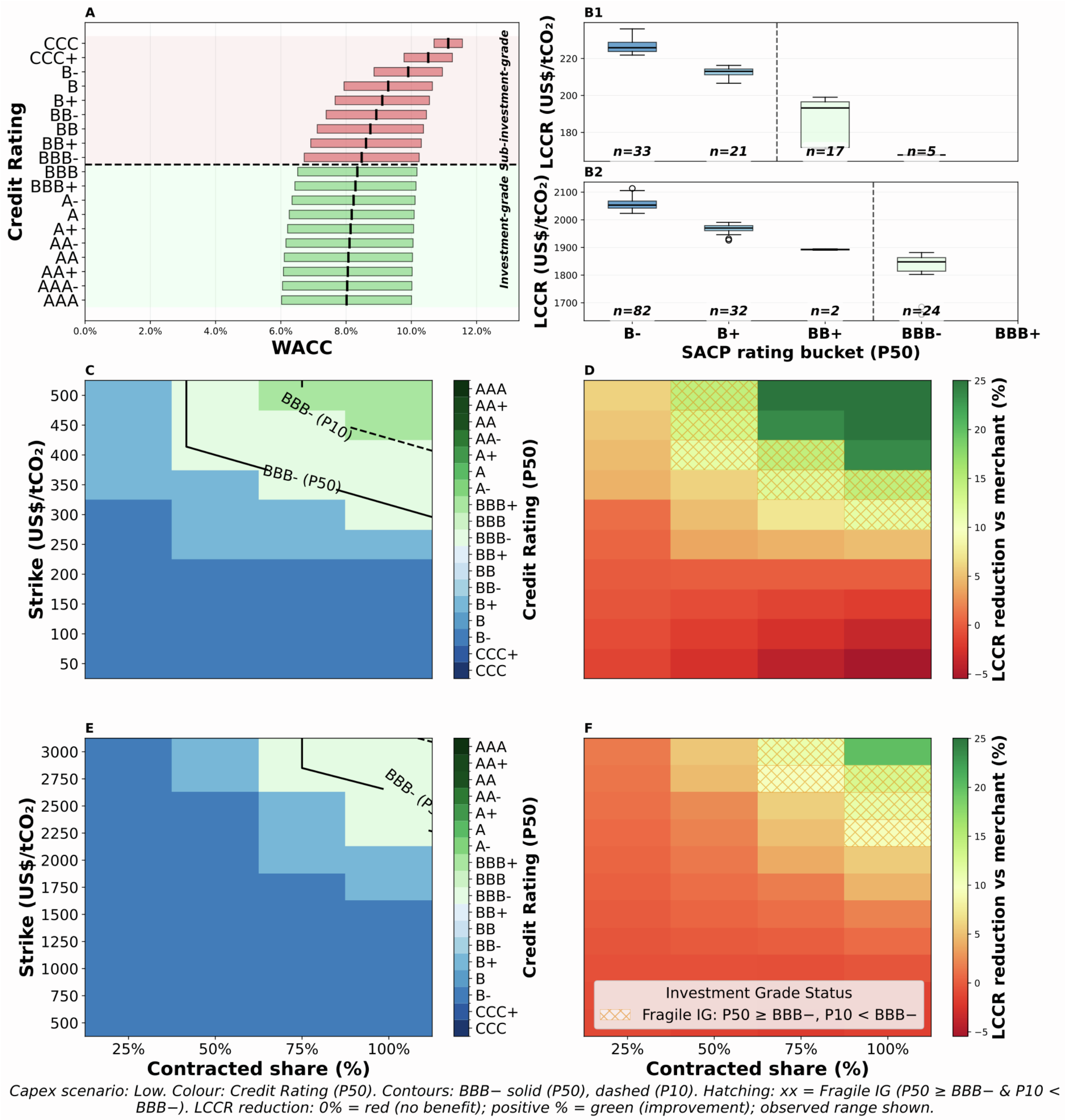

Figure S5. LCCR heatmaps at lower capital cost for CDR offtake and coverage levels, Related to Figure 4. *(a) cost of capital by credit rating (b) LCCR distributions by credit rating, shown for BECCS (B1) and DAC (B2); boxes span the interquartile range with the line at the median, whiskers extend to 1.5xIQR, points beyond are plotted individually, and n is the number of scenarios in each rating bucket (c) Rating heat maps for BECCS (d) LCCR change compared to benchmark for BECCS (e) Rating heat maps for DAC (f) LCCR change compared to benchmark for DAC. The colour reports LCCR scale.*

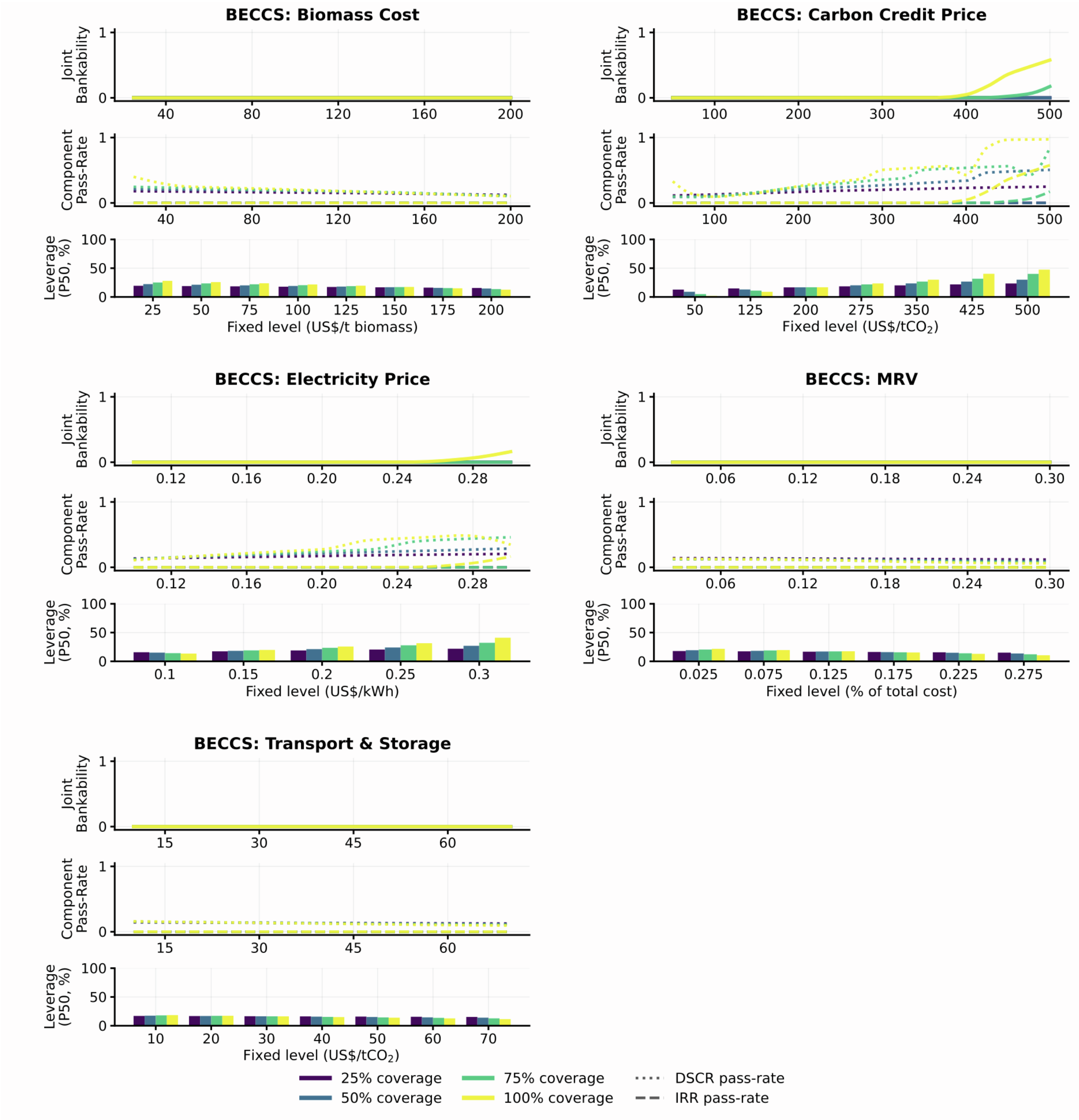

Figure S6. Bankability curves for BECCS under high capital cost scenario, Related to Figure 3.

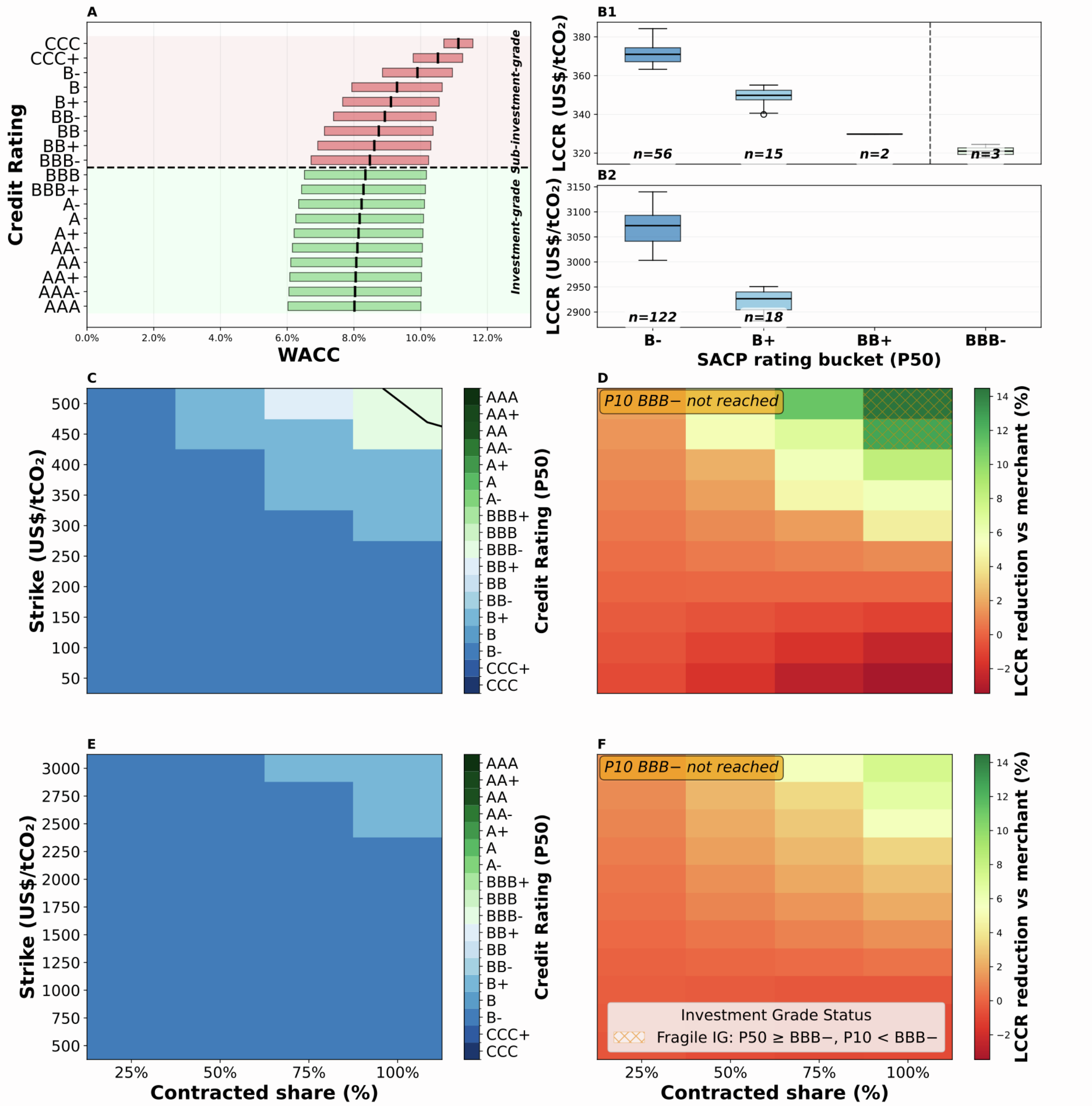

Capex scenario: High. Colour: Credit Rating (P50). Contours: BBB− solid (P50), dashed (P10). Hatching: xx = Fragile IG (P50 ≥ BBB− & P10 < BBB−). LCCR reduction: 0% = red (no benefit); positive % = green (improvement); observed range shown.

Figure S7. LCCR heatmaps at higher capital cost for CDR offtake and coverage levels, Related to Figure 4.

(a) cost of capital by credit rating (b) LCCR distributions by credit rating, shown for BECCS (B1) and DAC (B2); boxes span the interquartile range with the line at the median, whiskers extend to 1.5xIQR, points beyond are plotted individually, and n is the number of scenarios in each rating bucket (c) Rating heat maps for BECCS (d) LCCR change compared to benchmark for BECCS (e) Rating heat maps for DAC (f) LCCR change compared to benchmark for DAC. The colour reports LCCR scale.

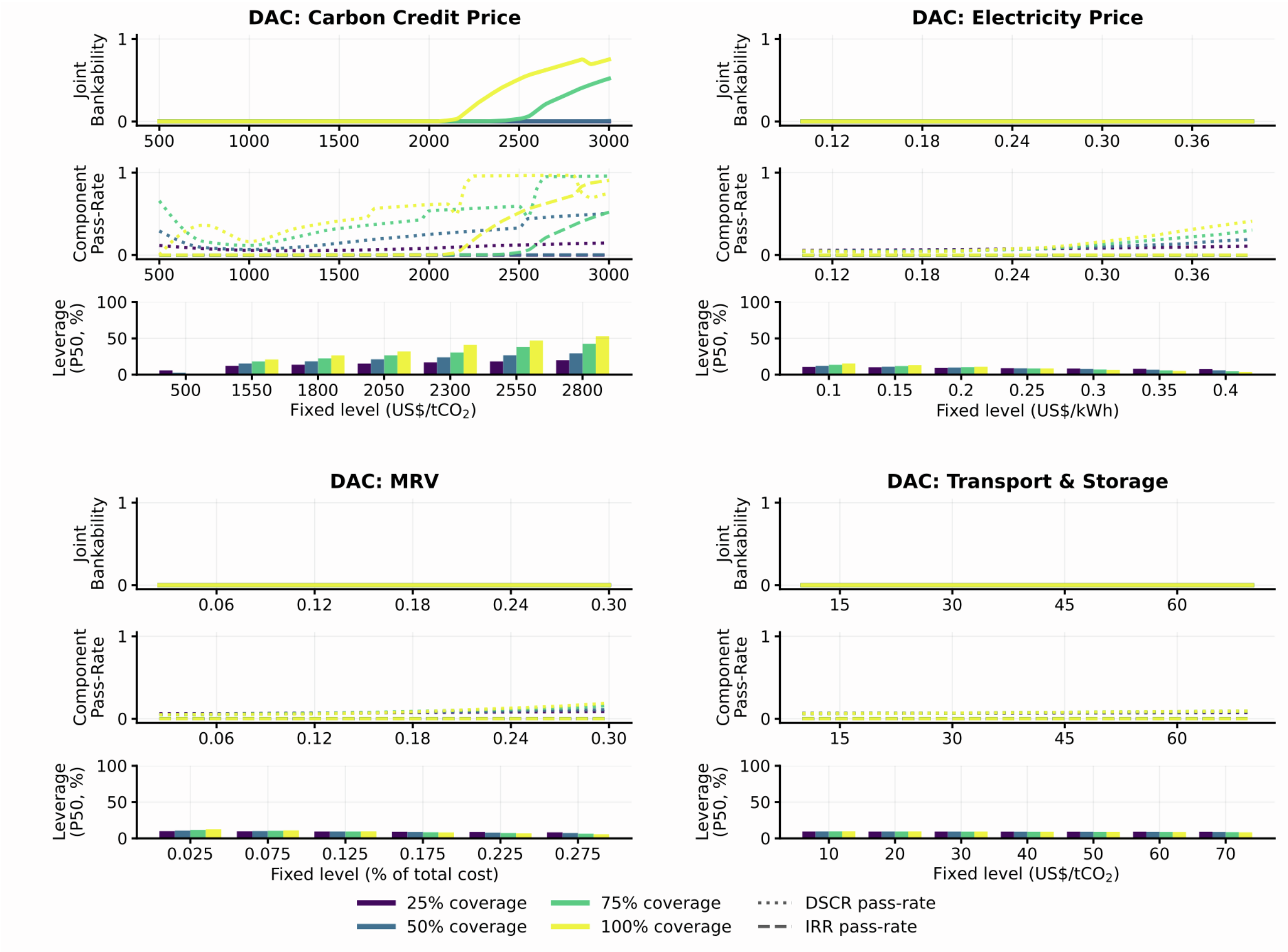

Figure S8. Bankability curves for DAC under low capital cost scenario, Related to Figure 3.

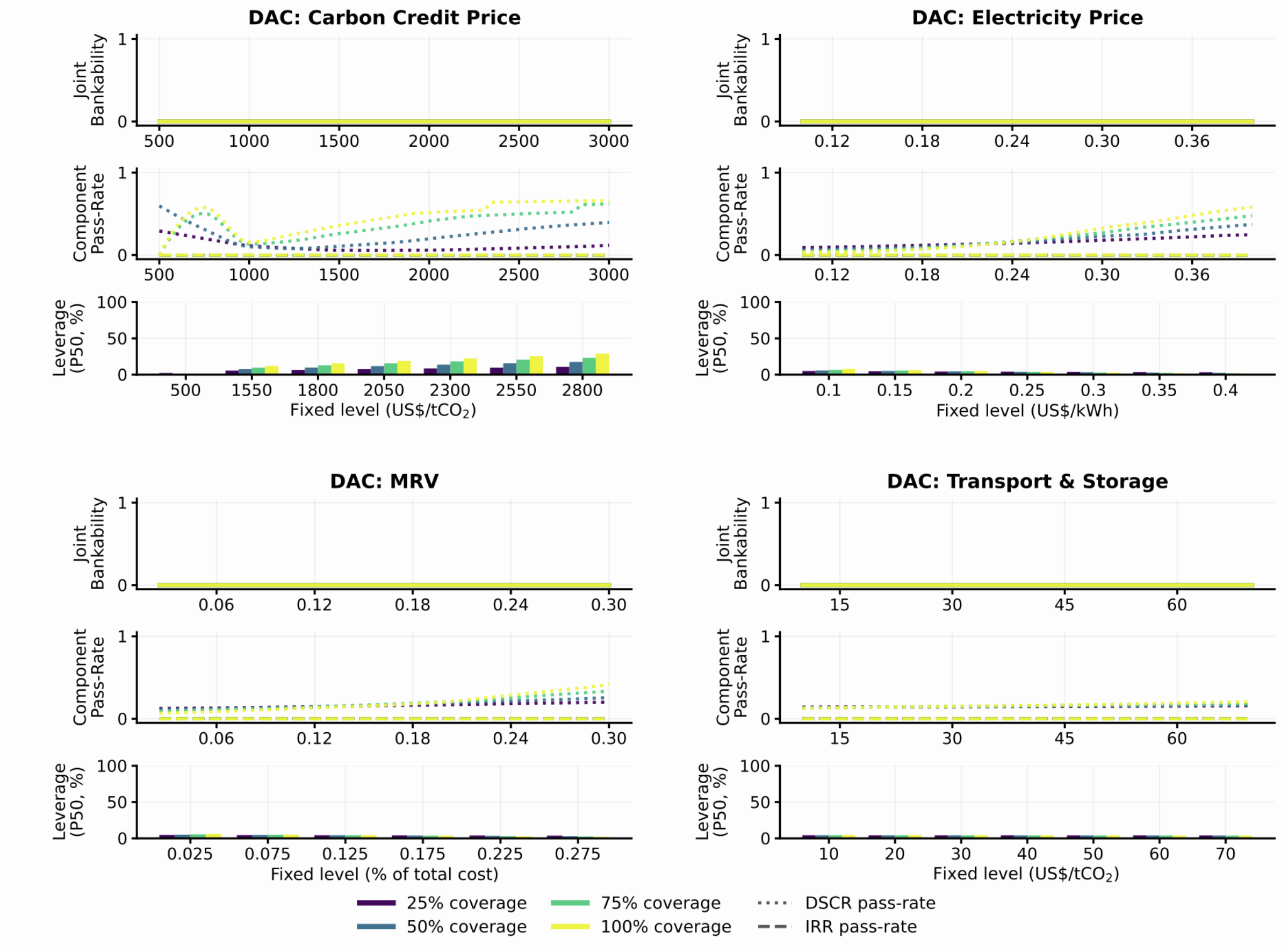

Figure S9. Bankability curves for DAC under high capital cost scenario, Related to Figure 3.

REFERENCES

[S1] APMG International (2016). The APMG public-private partnership (PPP) certification guide (APMG International).

[S2] Cummins, K., Klembara, M., Thompson, A., Corriere, O., Goldfield, E., and Dean, W. (2024). Portfolio insights: learning from case studies: financing and development approaches from recent first-of-a-kind projects. Office of Clean Energy Demonstrations.

[S3] Wärnelid, R. (2015). Debt service reserve account (DSRA). Forvis Mazars Financ. Model. https://financialmodelling.forvismazars.com/resources/debt-service-reserve-account-dsra/.

[S4] Lutereau, P.F., Macchi, C., Nietvelt, K., Selting, A.C., Langberg, R.M., and Chayrigues, V. (2022). Sector-specific project finance rating methodology (S&P Global Ratings).

[S5] Lutereau, P.F., Macchi, C., Langberg, R.M., Merani, V.H., Nietvelt, K., and Chayrigues (2022). General project finance rating methodology (S&P Global Ratings).

[S6] Federal Reserve Bank of St. Louis (2025). ICE BofA US corporate index option-adjusted spread. https://fred.stlouisfed.org/series/BAMLC0A4CBBB.

[S7] Judge, A., and Korzhenitskaya, A. (2022). Do Credit Ratings Determine Capital Structure? Int. J. Econ. Bus. *29*, 89–118. https://doi.org/10.1080/13571516.2021.1961563.

[S8] Chiquier, S., Patrizio, P., Bui, M., Sunny, N., and Dowell, N.M. (2022). A comparative analysis of the efficiency, timing, and permanence of CO2 removal pathways. Energy Environ. Sci. *15*, 4389–4403. https://doi.org/10.1039/D2EE01021F.

[S9] Manasa, P., Sambasivam, S., and Ran, F. (2022). Recent progress on biomass waste derived activated carbon electrode materials for supercapacitors applications—A review. J. Energy Storage *54*, 105290. https://doi.org/10.1016/j.est.2022.105290.

[S10] International Renewable Energy Agency (2012). Renewable energy cost analysis: biomass for power generation (IRENA).

[S11]. Jenkins, B.M., Baxter, L.L., Miles, T.R., and Miles, T.R. (1998). Combustion properties of biomass. Fuel Process. Technol. *54*, 17–46. https://doi.org/10.1016/S0378-3820(97)00059-3.

[S12] Gustafsson, K., Sadegh-Vaziri, R., Grönkvist, S., Levihn, F., and Sundberg, C. (2021). BECCS with combined heat and power: assessing the energy penalty. Int. J. Greenh. Gas Control *108*, 103248. https://doi.org/10.1016/j.ijggc.2020.103248.

[S13] Menmuir, D., and Berry, K. (2022). Next generation carbon capture technology: technoeconomic analysis work package 6.

[S14] Mercer, L., Burke, J., and Rodway-Dyer, S. (2024). Towards improved cost estimates for monitoring, reporting and verification of carbon dioxide removal (Grantham Institute on Climate Change and the Environment).

[S15] Smith, E., Morris, J., Kheshgi, H., Teletzke, G., Herzog, H., and Paltsev, S. (2021). The cost of CO2 transport and storage in global integrated assessment modeling. Int. J. Greenh. Gas Control *109*, 103367. https://doi.org/10.1016/j.ijggc.2021.103367.

[S16] Wood pellet prices index for NW Europe | Argus Media https://www.argusmedia.com/en/methodology/key-commodity-prices/northwest-europe-wood-pellet.

[S17] Sovacool, B.K., and Ryu, H. (2025). Beyond economies of scale: learning from construction cost overrun risks and time delays in global energy infrastructure projects. Energy Res. Soc. Sci. *123*, 104057. https://doi.org/10.1016/j.erss.2025.104057.

[S18] CDR.fyi and OPIS (2025). Bridging the gap: Durable market pricing survey (OPIS: A Dow Jones Company).

[S19] CDR.fyi (2025). Price Index. https://www.cdr.fyi/.

[S20] Ofgem (2025). Wholesale market indicators. https://www.ofgem.gov.uk/news-and-insight/data/data-portal/wholesale-market-indicators.

[S21] Young, J., McQueen, N., Charalambous, C., Foteinis, S., Hawrot, O., Ojeda, M., Pilorgé, H., Andresen, J., Psarras, P., Renforth, P., et al. (2023). The cost of direct air capture and storage can be reduced via strategic deployment but is unlikely to fall below stated cost targets. One Earth *6*, 899–917. https://doi.org/10.1016/j.oneear.2023.06.004.

[S22] Orbuch, R. (2020). Stripe's first carbon removal purchases. https://stripe.com/blog/first-negative-emissions-purchases.

[S23] Stripe (2021). Stripe commits $8M to six new carbon removal companies. https://stripe.com/gb/newsroom/news/spring-21-carbon-removal-purchases.

[S24] Department for Energy Security and Net Zero (2025). International non-domestic energy prices. GOV.UK. https://www.gov.uk/government/statistical-data-sets/international-industrial-energy-prices.

[S25] Smith, S., Fuss, S., Buck, H., Schenuit, F., Pongratz, J., Schulte, I., Lamb, W.F., Probst, B., Edwards, M., Nemet, G.F., et al. (2024). The State of Carbon Dioxide Removal - 2nd Edition. https://doi.org/10.17605/OSF.IO/F85QJ.

[S26] van der Spek, M., Bardow, A., Baum, C.M., Bolongaro, V., Dufour-Décieux, V., Esch, C., Fritz, L., Garcia, S., Hamann, C., Hondeborg, D., et al. (2025). An ecosystem of carbon dioxide removal reviews – part 1: direct air CO2 capture and storage. Energy Environ. Sci. *18*, 9713–9785. https://doi.org/10.1039/D5EE01732G.

[S27] Almaraz, O., Haque, M.E., Bhattacharyya, D., Liu, X., and Palanki, S. (2025). Modular integrated system for carbon-neutral methanol synthesis using direct air capture and carbon-free hydrogen production. Ind. Eng. Chem. Res. *64*, 22775–22789. https://doi.org/10.1021/acs.iecr.5c02311.

[S28] Bisotti, F., Hoff, K.A., Mathisen, A., and Hovland, J. (2024). Direct air capture (DAC) deployment: a review of the industrial deployment. Chem. Eng. Sci. *283*, 119416. https://doi.org/10.1016/j.ces.2023.119416.

[S29] Kato, E., and Yamagata, Y. (2014). BECCS capability of dedicated bioenergy crops under a future land-use scenario targeting net negative carbon emissions. Earths Future *2*, 421–439. https://doi.org/10.1002/2014EF000249.

[S30] Möllersten, K. (2022). Assessment of classes of CDR methods: Technology Readiness, Costs, Impacts and Practical Limitations of Biochar as Soil Additive and BECCS. In.

[S31] Zhang, D., Bui, M., Fajardy, M., Patrizio, P., Kraxner, F., and Mac Dowell, N. (2020). Unlocking the potential of BECCS with indigenous sources of biomass at a national scale. Sustain. Energy Fuels *4*, 226–253. https://doi.org/10.1039/C9SE00609E.

[S32] Bui, M., Adjiman, C.S., Bardow, A., Anthony, E.J., Boston, A., Brown, S., Fennell, P.S., Fuss, S., Galindo, A., Hackett, L.A., et al. (2018). Carbon capture and storage (CCS): the way forward. Energy Environ. Sci. *11*, 1062–1176. https://doi.org/10.1039/C7EE02342A.

[S33] Fajardy, M., Köberle, D.A., Mac Dowell, N., and Fantuzzi, A. (2019). BECCS deployment: a reality check. Grantham Institute (Grantham Institute).

[S34] Fajardy, M., Chiquier, S., and Dowell, N.M. (2018). Investigating the BECCS resource nexus: delivering sustainable negative emissions. Energy Environ. Sci. *11*, 3408–3430. https://doi.org/10.1039/C8EE01676C.

[S35] McQueen, N., Gomes, K.V., McCormick, C., Blumanthal, K., Pisciotta, M., and Wilcox, J. (2021). A review of direct air capture (DAC): scaling up commercial technologies and innovating for the future. Prog. Energy *3*, 032001. https://doi.org/10.1088/2516-1083/abf1ce.

[S36] Block, S., Viebahn, P., and Jungbluth, C. (2024). Analysing direct air capture for enabling negative emissions in Germany: an assessment of the resource requirements and costs of a potential rollout in 2045. Front. Clim.
